# Electrochemical CO_2_ reduction toward multicarbon alcohols - The microscopic world of catalysts & process conditions

**DOI:** 10.1016/j.isci.2022.104010

**Published:** 2022-03-03

**Authors:** Theresa Jaster, Alina Gawel, Daniel Siegmund, Johannes Holzmann, Heiko Lohmann, Elias Klemm, Ulf-Peter Apfel

**Affiliations:** 1 Department of Energy, Fraunhofer Institute for Environmental, Safety, and Energy Technology UMSICHT, Osterfelder Str. 3, D46047 Oberhausen, Germany; 2 Inorganic Chemistry I, Ruhr University Bochum, Universitätsstr. 150, D44801 Bochum, Germany; 3 Institute of Chemical Technology, University of Stuttgart, Pfaffenwaldring 55, D70569 Stuttgart, Germany

**Keywords:** Catalysis, Electrochemistry

## Abstract

Tackling climate change is one of the undoubtedly most important challenges at the present time. This review deals mainly with the chemical aspects of the current status for converting the greenhouse gas CO_2_ via electrochemical CO_2_ reduction reaction (CO_2_RR) to multicarbon alcohols as valuable products. Feasible reaction routes are presented, as well as catalyst synthesis methods such as electrodeposition, precipitation, or sputtering. In addition, a comprehensive overview of the currently achievable selectivities for multicarbon alcohols in CO_2_RR is given. It is also outlined to what extent, for example, modifications of the catalyst surfaces or the use of bifunctional compounds the product distribution is shifted. In addition, the influence of varying electrolyte, temperature, and pressure is described and discussed.

## Graphical abstract

**Figure:**
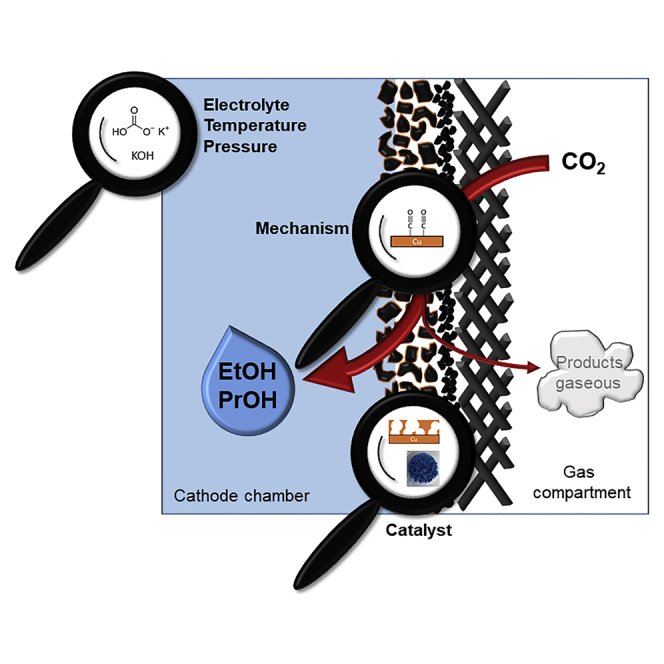

## Introduction

The progressive climate change is a globally relevant issue, and accordingly to the Intergovernmental Panel on Climate Change (IPCC) reports, humanity has so far caused a rise in global temperature of about 1°C. To avoid drastic adverse effects on biodiversity, the melting of ice caps, and further rise of sea levels, a value of 1.5°C related to the pre-industrial level should not be exceeded (Abram et al., 2019). Regarding the potent greenhouse gas CO_2_, the average global atmospheric concentration exceeded 400 ppm in 2016, which is the highest level ever recorded. In addition, the worldwide fossil fuel emissions of CO_2_ increased by more than 2% in 2018 (Abram et al., 2019). Therefore, strategies are needed that prevent further increase of the CO_2_ concentration in our atmosphere. The options discussed in this context are the capturing of carbon dioxide as well as its direct conversion. Along this line, the electrochemical reduction of CO_2_ via appropriate catalysts into value-added products is a promising strategy. The obtainable products include C_1_ compounds, such as CO, CH_4_, HCOOH, or CH_3_OH, as well as multicarbon products, e.g. acetic acid and ethylene, and C_2+_ alcohols (ethanol and propanol). This review focusses on the formation of multicarbon alcohols to summarize recent developments, which moved the electrochemical CO_2_ reaction a few steps closer to an industrial realization. Multicarbon alcohols are important target products in electrochemical CO_2_RR, as they are valuable basic chemicals for the chemical industry, can be used for energy production or as fuel additives (Jouny et al., 2018). An additional route for ethanol production thus further diversifies the feedstock for various products.

With regard to the current state of the art, only CO and HCOOH are commercially viable. However, market analyses show that by further development of catalysts, electrodes, and cells and with it a consequent reduction in energy and product separation costs, higher alcohols are promising products for the future (Jouny et al., 2018; Somoza-Tornos et al., 2021). They have a larger market potential than CO and formic acid (Jouny et al., 2018). According to Jiao and coworkers, a yield of at least 62% should be achieved for n-propanol, and 77% for ethanol at −0.7 V to become economically feasible. The current densities should be in the range of 200–400 mA cm^−2^ (Jouny et al., 2018). A more detailed techno-economic analysis of the CO_2_RR products can be found in Review *Electrochemical CO_2_* *reduction - The macroscopic world of electrode design, reactor concepts & economic aspects*.

Multicarbon alcohols are formed during carbon dioxide reduction reaction (CO_2_RR) according to the following reaction equations:

$${2\text{CO}}_{2}+{9\text{H}}_{2}\text{O}+{12\text{e}}^{-}\to {\text{CH}}_{3}{\text{CH}}_{2}\text{OH}+{12\text{OH}}^{-}$$

$${3\text{CO}}_{2}+{13\text{H}}_{2}{\text{O}+18\text{e}}^{-}\to {\text{CH}}_{3}{\text{CH}}_{2}{\text{CH}}_{2}\text{OH}+{18\text{OH}}^{-}$$

Both the mechanism for the formation of C_2+_ alcohols and catalysts that enable the selective electrocatalytic CO_2_RR to C_2+_ alcohols are considered. Furthermore, the influence of process conditions and techno-economic considerations are also explained in more detail.

In addition, it can be highly effective to couple electrochemical CO_2_ reduction with biocatalyzed methods. Schmid and coworkers achieved an FE of almost 100% for the conversion of CO_2_ to butanol and hexanol with a fermentation following the CO_2_RR using bacterium *Clostridium autoethanogenum* and *C*. *kluyveri* (Haas et al., 2018).

In terms of the electrocatalytic conversion, different types of electrolysers are described in literature. Basically, they can be divided into three main types: liquid-phase, gas-phase, and solid-oxide electrolyser cell (Kibria et al., 2019). The oxygen evolution reaction (OER) usually takes place at the anode of the electrolyzers and the CO_2_RR at the cathode. One ubiquitous and dominating problem with CO_2_RR in general is the competing, parasitic reduction of water to H_2_ (hydrogen evolution reaction, HER) (Lv et al., 2018b; Albo et al., 2019; Gabardo et al., 2019; Gao et al., 2019; Martić et al., 2019, 2020; Xiang et al., 2019; Chang et al., 2020; Dutta et al., 2020; Kim et al., 2020b; Song et al., 2020; Wei et al., 2020; Zhang et al., 2020c; Herzog et al., 2021; Wang et al., 2021), which occurs in the same potential range as the CO_2_ reduction. Thereby, the Faraday efficiency for the formation of hydrogen in CO_2_RR with the target product ethanol is typically reported to be above 30% (Kim et al., 2020b). However, especially for the CO_2_RR to higher alcohols, the preferential formation of ethylene is a further problem and numerous studies focus on the selectivity inversion between ethylene and ethanol (Gu et al., 2021; Kim et al., 2021; Santatiwongchai et al., 2021; Wang et al., 2021).

To indicate the selectivity of a catalyst or electrode, the so-called Faraday efficiency (FE, Equation 1) is given by

(Equation 1) $$FE=\;\frac{z\;\text{·}n\text{·}F}{I\;\text{·}t\;}\;\text{·}100$$

(*z* - number of electrons transferred; *n* - amount of substance of product; *F* - Faraday constant, *I* - current applied; *t* - reaction time).

Particularly for studies that focus on catalyst design and synthesis, H-type cells are widespread, despite the severe limitations of those systems (Burdyny and Smith, 2019). Its name is derived from its H-like form with cathode and anode compartments filled with liquid electrolyte, separated via an ion exchange membrane. The catalyst is usually deposited on glassy carbon or carbon paper and the CO_2_ is dissolved in the electrolyte. While this setup allows for simple and rapid testing of catalysts, it suffers from mass-transport limitation due to the low solubility of CO_2_ and can due to carbonate formation not be operated with alkaline electrolytes like KOH, which have been shown to improve CO_2_RR activity and C_2+_ selectivity (Carroll et al., 1991; Kibria et al., 2019). The low CO_2_ solubility and therefore availability limits the maximum current densities in H-type cells to about 100 mA cm^−2^, rendering them not feasible to be used in industrial CO_2_RR processes (Weekes et al., 2018). However, as the local conditions and, thus, selectivity are highly dependent on the current density and potential applied, the results obtained in an H-type cell make it difficult to draw significant conclusions about the catalyst performance under industrially relevant conditions. Those limitations demand the use of alternative setups for testing and optimizing of catalysts under realistic conditions at higher current densities (Weekes et al., 2018; Burdyny and Smith, 2019). This means that catalyst testing should be carried out under reasonable conditions like current densities of at least 200 mA cm^−2^ and stability tests of the catalysts and electrodes used of at least 24 h (Burdyny and Smith, 2019; Martić et al., 2019; Siegmund et al., 2021).

Therefore, flow cells or gas-phase electrolysers (by using membrane electrode assemblies) should be used, in which gas and electrolyte streams are continuously supplied and cycled, respectively, to achieve the industrially relevant current density of >200 mA cm^−2^ (Weekes et al., 2018; Li et al., 2019b; Martić et al., 2019).

## Mechanistic principles and catalyst design for CO_2_ reduction to multicarbon alcohols

The following brief overview describes the mechanistic background of the formation of multicarbon alcohols during the electrochemical CO_2_ reduction.

In addition to general considerations on the mechanism of C-C coupling at the beginning of the chapter, various mechanisms found for diverse catalysts are further presented with only few catalysts being addressed here as examples. A more detailed discussion of the different catalysts and their operating principles is given in chapters "Structural properties and crystal orientations" ff. For CO_2_RR, copper plays a special role here, because it can form a variety of products and is the only metal capable of forming higher hydrocarbons and oxygenates. The diversity of possible products obtained by copper catalysts illustrates the complexity of the reduction reaction (Hori, 2008; Kuhl et al., 2012; Nitopi et al., 2019). For systematic optimization, a comprehensive understanding of the underlying reaction mechanism is fundamental.

### General mechanistic considerations

**Figure 1:**
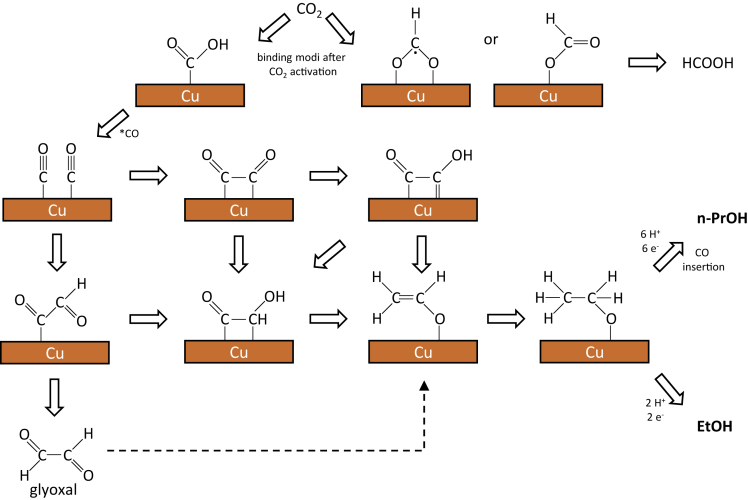
Visualization of possible CO2 reduction pathways with ethanol and propanol as the target products Own representation based on Liu et al., 2019; Hasani et al., 2020; Todorova et al., 2020 and Cheng et al., 2021.

In the electrochemical CO_2_ reduction process, an initial electrochemical transfer of H^+^/e^−^ to CO_2_ occurs. The resulting intermediate can bind to the electrode surface either via oxygen or via carbon. In the former case, formation of HCOOH can be expected, whereas in the latter case CO (Figure 1) is obtained, making this step crucial for the formation of the products in CO_2_RR (Cheng et al., 2016; Feaster et al., 2017; Chernyshova et al., 2018). Thereby, CO is widely considered as a key intermediate for further reduced C_1_ and C_2_ products, supported by investigations on the reduction of CO as well as *in situ* measurements (Hori et al., 1994, 1997; Wuttig et al., 2016; Gunathunge et al., 2017; Pérez-Gallent et al., 2017b; Bertheussen et al., 2018; Birdja et al., 2019; Nitopi et al., 2019).

The C-C bond formation is the crucial reaction step that separates the pathways for single and multicarbon products. The dimerization of two ∗CO species is commonly considered a key step for the C-C bond formation, resulting in bidentate ∗CO∗CO as intermediate species. Figure 1 shows the proposed mechanistic pathway for the formation of ethanol and n-propanol (Cheng et al., 2021). The subsequent reduction steps to ∗CO∗CHOH or ∗CO∗COH have been considered as possible follow up intermediates (Calle-Vallejo and Koper, 2013; Kortlever et al., 2015; Montoya et al., 2015; Goodpaster et al., 2016; Cheng et al., 2017, 2021; Garza et al., 2018; Hanselman et al., 2018; Jiang et al., 2018; Todorova et al., 2020). Herein, the ∗COCOH intermediate could be observed via *in situ* IR spectroscopy (Pérez-Gallent et al., 2017a). Furthermore, operando Raman spectroscopy results suggest that the dimerization of ∗CO is competing with the hydrogenation to ∗COH or ∗CHO, which are further reduced to C_1_ products (Todorova et al., 2020). Along this line, C-C coupling steps via reaction of ∗CHO or ∗COH with CO to ∗COCHO or ∗COCOH also have been postulated (Goodpaster et al., 2016; Xiao et al., 2016; Garza et al., 2018; Jiang et al., 2018). Methylcarbonyl represents the most likely intermediate where a distinction takes place as to whether hydrogenation to ethanol or acetaldehyde occurs or whether further coupling with ∗CO and thus the formation of propanol takes place. In this case, the ∗CO attacks the carbonyl carbon of the acetaldehyde (Chang et al., 2020).

### Influence of Cu catalyst (surface) properties on selectivity

Notably, the structure and properties of the (copper) electrodes have a significant influence on the C-C coupling step (Gao et al., 2019; Fan et al., 2020). It has been shown that the selectivity of CO_2_RR is dependent on the exposed copper facets. For example, Cu(110) and Cu(551) facets promote the formation of C_2_ products (Hori et al., 2002; Schouten et al., 2012, 2013; Kim et al., 2016). Engineering of catalyst size and morphology has been proven successful in steering the selectivity toward C_2_ products due to the exposed facets and differences in surface features like defect density, grain boundaries, and overall surface. Furthermore, various studies showed that morphological changes of the catalysts under the chosen process conditions have a significant effect on the product selectivity (Gregorio et al., 2020; Hou et al., 2020). In particular, too large particles as well as high current densities were identified as crucial parameters leading to aggregation and consequently to an altered product selectivity (Huang et al., 2018). The influence of structure on the pursued reaction mechanism was investigated for oxide-derived (OD) copper. It was shown that step square sites (s-sq) support the formation of C_2+_ alcohols, due to favorable thermodynamics for hydrogenation. In addition, the bond length between CO and the active site was correlated with the observed preferential product formation. For example, ethanol is preferentially formed at s-sq sites, which have the shortest determined bond length of 1.296 Å compared to planar-square and concave square, where ethylene formation preferentially occurs (Cheng et al., 2021). Another way of tuning catalyst selectivity is by adjusting the copper oxidation state. While the increased selectivity and activity of oxide-derived materials has partially been assigned to morphologic effects resulting from the reduction, results indicate that Cu^+^ and subsurface oxygen species play a role, too (Mistry et al., 2016; Favaro et al., 2017; Xiao et al., 2017b; Luna et al., 2018; Pander et al., 2018; Zhou et al., 2018). Recent results show that for copper-oxide-containing electrodes, reduction of the oxide layer occurs first before product formation due to CO_2_RR and HER (Löffler et al., 2021). The difference with pure copper electrodes is that the reduction of the oxide leads to the increased occurrence of defects and grain boundaries, resulting in a highly active surface.

After C-C coupling, subsequent reduction steps lead to the multicarbon reduction products ethylene and ethanol. The possible intermediates and conceivable branching in the mechanistic pathway are, however, still under debate (Todorova et al., 2020). Bell and coworkers described ∗COCHO as first dimer intermediate followed by reduction to either glyoxal or ∗CO∗CHOH, and depending on the products formed, the reaction pathway proceeds either ethanol or ethylene, respectively. Glyoxal is subsequently reduced to acetaldehyde and ethanol (Garza et al., 2018). Acetaldehyde has been confirmed as an important intermediate toward ethanol formation via *in situ* NMR spectroscopy as well as mass spectrometry (Bertheussen et al., 2016; Clark and Bell, 2018). Other authors describe (as also can be seen in Figure 1) ∗CO∗COH as the key coupling product, whereby the mechanism then follows a different path via the reduction to ∗CCO. According to Goddard and coworkers, the next intermediate ∗CH∗COH is either dehydrated to form ∗CH∗C, which yields ethylene after another hydrogenation step, or to ∗CHCHOH, which is converted to ethanol via three further hydrogenation steps (Cheng et al., 2017; Xiao et al., 2017a). According to Calle-Vallejo and coworkers, acetaldehyde is the selectivity determining intermediate, which is converted to either ethylene or ethanol after further reaction steps (Calle-Vallejo and Koper, 2013; Hanselman et al., 2018). Contrarily, Asthagiri and coworkers postulated acetaldehyde and the two further hydrogenated species ∗CH_2_CH_2_O∗ and CH_3_CH_2_O∗ as three possible points where the pathways diverge (Luo et al., 2016). Hirunsit and coworkers mention the dissociation of the C-O bonds as most important for following the pathway either toward ethanol or ethylene formation (Santatiwongchai et al., 2021). Investigations on Cu(100) surfaces have shown that the protonation steps five to seven are decisive and if the C-O bond is about to break later, EtOH will be formed instead of ethylene. To conclude, this work shows that the following intermediates lead to ethanol: ∗CH_3_CO, ∗CH_3_CHO, ∗CH_3_CHOH, and ∗CH_3_CH_2_O whereas ∗CH_2_CH, ∗CCH_2,_ and ∗CHCH lead to ethylene. ∗CHCHOH, ∗CH_2_CHO, ∗HOCH_2_CH_2_O, ∗CH_2_CH_2_OH, ∗CH_2_CHOH, and ∗HOCH_2_CH_2_OH are the intermediates which can result in either ethanol or ethylene formation.

### Multimetallic and bifunctional catalysts

**Figure 2:**
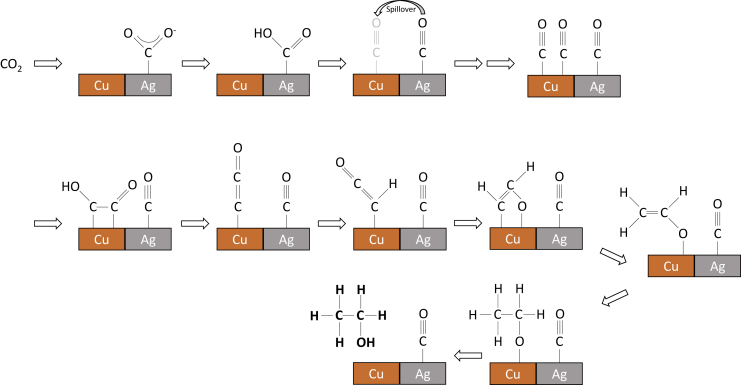
Proposed mechanism for CO_2_RR to CO, followed by ethanol formation at bimetallic Cu-Ag foam Own representation based on Dutta et al., 2020.

**Figure 3:**
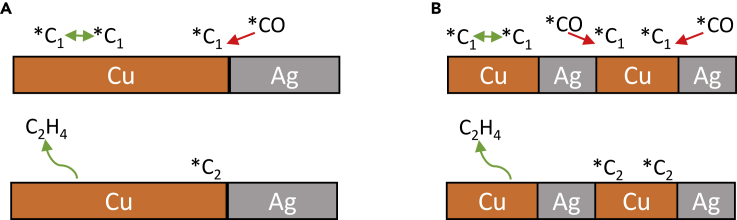
Schematic hypothetical representation of CO insertion on a Cu/Ag catalyst and the influence of domain size on product distributions (A) With large domains of Cu/Ag catalysts and consequently low amount of biphasic boundaries. (B) With smaller domains of Cu/Ag catalysts and correspondingly pronounced biphasic boundaries, which favor C-C coupling. The target product ethanol is highlighted as ∗C_2_. Own representation based on Lee et al., 2017.

To increase the selectivity toward multicarbon alcohols, multimetallic catalysts are frequently used. For example, the ethanol to ethylene ratio could be increased by a factor of 12.5 by introducing zinc as a co-catalyst to copper (Ren et al., 2016). This is where the so-called spillover effect occurs. The effect was described not only for Cu-Zn (Ren et al., 2016) but also for Cu-Ag (Dutta et al., 2020; Martić et al., 2020; Ting et al., 2020), Cu-Pd (Rahaman et al., 2020), and for catalysts with Cu nanoparticles and pyridinic nitrogen in N-doped carbon (Han et al., 2020a). One of the mechanisms proposed for bimetallic catalysts is shown in Figure 2. In this process, CO_2_ is reduced to CO at Zn, Ag, Pd, or pyridinic N sites, where CO is only weakly adsorbed (Ren et al., 2016; Han et al., 2020a; Rahaman et al., 2020) and CO migration to active copper sites can be achieved. There, CO is bound superiorly and will either be further reduced or undergo further reactions with adjacent ∗C1 and ∗C2 intermediates (Han et al., 2020a). With respect to the Cu-Ag-containing catalysts, the ratio of Cu: Ag is expected to have a direct influence on the product distribution due to an altered electronic structure (Martić et al., 2020). The interaction of copper and silver results in a shift of the E_d_ value, which represents the location of the center of the d-band, from that of copper at-3.30 eV by −0.56 eV toward that of silver (−5.36 eV). The electronic change results in less binding of CO_2_RR and HER intermediates, leading to preferential CO formation with FEs ranging from 55% to 68%. The main liquid product was ethanol with about 25% FE at 400 mA cm^−2^. Furthermore, the selectivity of 34.2% for ethanol in phase-blended Ag-Cu catalysts has been shown to be three times higher than with pure Cu_2_O (Lee et al., 2017). The authors emphasized the importance of the biphasic boundary for improved ethanol to ethylene selectivity. Upon modification of the distance between CO-producing Ag and Cu sites, increased insertion of CO and consequently formation of EtOH (demonstrated by ∗C_2_) can be achieved (Figure 3).

Ag-Cu foams could be activated for ethanol production via a 12 h thermal annealing in air at 200°C. The obtained oxide-derived bimetallic catalyst showed a maximum FE of 33.7% for ethanol at −1.0 V and 6.9% for propanol at −0.9 V vs. RHE, while the formation of those products was negligible without the mentioned thermal treatment of the catalyst (Dutta et al., 2020).

In addition to the spillover effect in bimetallic compounds, the combination of Cu nanorods (nr) and NGQ (nitrogen-doped graphene quantum dots) also enables an interesting mechanism. Oxygenated C_2_ intermediates were stabilized at the NGQ/Cu-nr, and by allowing both Cu-nr and NGQ to form C_2_ products, the formation of the multicarbon products is promoted by dual active sites. On both components, the existence of ∗CO as intermediate could be detected, but there was no evidence for a spillover or tandem effects (Chen et al., 2020a). Both effects describe the same process from a different point of view. However, while the term spillover effect describes the adsorption of the CO formed and its migration on the catalyst surface, the term tandem effect refers to the catalyst, i.e. that it has different domains on which different reaction steps take place. Therefore, a dual active-site mechanism was suggested, indicating the presence of active sites in NGQ as well as in Cu for the formation of C_2+_ products. In addition, the catalyst was found to stabilize the intermediate ∗CH_2_∗CHO, which is crucial for the higher FEs (52.4%) of multicarbon alcohols.

### N- and P-doped catalysts

Heteroatom-doped nanostructured carbon materials have also been examined as catalysts for the reduction of CO_2_ to alcohols. Their performance can be tuned via the nature and amount of heteroatom sites as well as the carbon morphology (Wu et al., 2019). A nitrogen- and boron-doped nano diamond catalyst reached a high ethanol selectivity of 93.2% at −1.0 V vs. RHE due to the synergistic effects of the heteroatom sites. The measurements were performed in H-type cells, with a CO_2_-saturated 0.1 M NaHCO_3_ electrolyte, and the total current densities were below 2 mA cm^−2^ (Liu et al., 2017). Because boron has an electron-poor p-orbital, it acts similarly to transition metals with an empty d-orbital and thus represents an active site for adsorption and subsequent reduction of CO as well as for CO_2_ (Zhu et al., 2021). For nitrogen-doped porous carbons, the high ethanol selectivity of 77% and 78% at −0.56 V vs. RHE has been attributed to synergistic effects between the carbon structure and active sites (Song et al., 2017, 2020). In addition, P-doping of catalysts could be used to adjust the adsorption strength for the CO intermediate. Thus, with P-doping, 2.8 times as much ethanol (15%) could be obtained with Cu_0.92_P_0.08_ C_2+_ product yield (Kong et al., 2021). Likewise, catalysts combining doped nanocarbons and copper catalysts have been described, reporting, e.g. tandem effects of heteroatom and metal sites with up to 64.8% FE for ethanol and 8.7% for propanol at −1.05 V vs. RHE (Song et al., 2016; Karapinar et al., 2019; Han et al., 2020a). However, it must be emphasized that the FEs of over 60% for ethanol achieved herein by different groups obtained under conditions of extremely low current densities between 2 and 16 mA cm^−2^. Hence, further improvements in systems allowing for higher current densities above 200 mA cm^−2^ are required to establish an industrial relevant process.

### Considerations about mechanistic understanding of CO_2_RR

In general, many mechanistic insights are obtained using computational methods such as DFT. Here, DFT is often used to show potential pathways for a target-oriented catalyst design and can reveal mechanistic information, e.g. regarding detailed reaction pathways (Li et al., 2020; Malkani et al., 2020; Santatiwongchai et al., 2021). The use of *in situ* techniques such as isotope labeling or the application of *in situ* spectroscopy such as XAS (X-ray absorption spectroscopy) or surface-enhanced vibrational spectroscopy methods can further help to complete the mechanistic understanding (Pérez-Gallent et al., 2017a; Malkani et al., 2020; Wang et al., 2020b). Studies of surface reconstruction in copper electrodes during CO_2_RR were e.g. conducted in 2017 by Waegele and coworkers as well as Koper and coworkers using Raman spectroscopy and Fourier transform infrared spectroscopy (FT-IR) (Gunathunge et al., 2017; Pérez-Gallent et al., 2017a). Furthermore, XAS has already been used to study the electronic as well as the coordinative structure on Cu catalysts during ongoing CO_2_RR (Xu et al., 2020; Herzog et al., 2021). Xu and coworkers describe in detail the advantages that *in situ* techniques offer, such as identifying the metals that provide the adsorption sites in the electrocatalytic reaction and analyzing metal-adsorbate interactions (Malkani et al., 2020). This contributes to a broader understanding of the mechanistic processes involved in CO_2_RR and for a more in-depth discussion on these techniques we refer to such papers.

## Catalysts

### Overview of current catalyst development

Table 1 provides an overview of recent developments in CO_2_RR to multicarbon alcohols, including the catalysts and electrolytes used as well as the resulting Faraday efficiencies. Firstly, copper and copper oxide as well as copper-oxide-derived (OD) catalysts are listed, followed by copper-carbon catalysts as well as copper catalysts, which were doped e. g. with boron or modified with halides, catalysts made of copper and another metal, and lastly miscellaneous catalysts, which do not fit in one of the categories mentioned before. The dominant usage of copper can be explained by its ability of producing multicarbon products during the reduction of CO_2_ (Loiudice et al., 2016; Garza et al., 2018; Karapinar et al., 2019; Malkhandi and Yeo, 2019; Jeong et al., 2020; Lei et al., 2020). The use of Cu electrodes in CO_2_ reduction experiments allows for the formation of a broad variety of products. Cyclic voltammetry (CV) measurements yielded CO, allyl alcohol, propionaldehyde, *n*-propanol, acetaldehyde, EtOH, ethylene, and methane in varying amounts and ratios (Clark and Bell, 2018). The table furthermore summarizes the FEs of the respective products. Thereby, it becomes visible that the selective formation of multicarbon alcohols still possesses a challenge. The products marked “C_2+_” usually contain high amounts of C_2_H_4_, which is often the main reason for the high overall FEs. This effect is a result of the fact that ethylene is generally preferred to ethanol formation in copper-based electrodes (Ren et al., 2016).

**Table 1:** Overview of recent development in the reduction of carbon dioxide to multicarbon alcohols (bold) between 2018 and 2021

Catalyst	Faraday Efficiency	Electrolyte	Potential/V vs RHE	Current densities/mA cm^.2^	Ref
**Copper and copper-oxide catalysts**					

Dendritic Cu	85.2% C_2+_ (35.5% C_2_H_4,_**38.0% EtOH**)	1 M KOH	–	800 (total)	(Xue et al., 2021)
Reconstructed porous Cu	80% C_2_	0.1 M KHCO_3_	−1.09	21 (C_2_ products)	(Han et al., 2020b)
CuO_x_	ca. 80% C_2+_	0.1 M CsHCO_3_	−0.9	∼8 (C_2+_ products	(Jeong et al., 2020)
Cu-oxide-/hydroxide-derived	ca. 70% C_2+_	0.1 M KHCO_3_	−1.05	40–50 (C_2+_ products)	(Lei et al., 2020)
Cu-NPs + polyaniline	80% C_2+_ with 40% C_2_H_4_, (EtOH, PrOH)	0.1 M KHCO_3_	−1.2	∼4 (total)	(Wei et al., 2020)
Multihollow Cu oxide	75.2% C_2+_	2 M KOH	−0.61	267 (C_2+_ products)	(Yang et al., 2020)
Cu(OH)_2_/Cu	25% C_2_H_4_ **5% EtOH**	0.1 M NaHCO_3_	−1.6	–	(Iijima et al., 2019)
Cu-Cu_4_O_3_	42% C_2_H_4_ **14% EtOH** **5% PrOH**	2.5 M KOH	−0.59	185 (C_2+_ products)	(Martić et al., 2019)
Cu@Cu_2_O	21% C_2_H_4_ **29% EtOH**	0.1 M KHCO_3_	−1.0	18 (total)	(Shang et al., 2019)
Cu_x_O	40% C_2_ (C_2_H_4_, EtOH)	2 M KOH	−1.17	234 (total)	(Xiang et al., 2019)
CuO_x_	69% C_2+_	0.1 M CsHCO_3_ + 0.1 M CsI	−1.0	46 (C_2+_ products)	(Gao et al., 2018)
Cu-NCs	60% C_2+_ (32% C_2_H_4_)	0.25 M KHCO_3_	−0.96	68 (total) 40 (C_2+_ products)	(Jiang et al., 2018)
CuCl-derived Cu	84% C_2+_ (>60% C_2_H_4_)	3 M KOH	−0.68	336 (C_2+_ products)	(Kibria et al., 2018)
Cu-NPs	62% C_2+_ (C_2_H_4_, EtOH, PrOH)	1 M KOH	−0.67	411 (C_2+_ products)	(Lv et al., 2018a)

**Copper-carbon catalysts**					

Nitrogen-doped graphene quantum dots on Cu-OD Cu nanorods	**52.4% C**_**2+**_**-Alcohols**	1 M KOH	−0.9	282 (total)	(Chen et al., 2020a)
Cu + N-C on PTFE- Substrate	**52% EtOH**	1 M KOH	−0.68	156 (EtOH)	(Wang et al., 2020b)
Cu-NPC	**64.6% EtOH** **8.7% PrOH**	0.2 M KHCO_3_	−1.05	∼8 (EtOH) ∼1.2 (PrOH)	(Han et al., 2020a)
Cu-C	**91% EtOH**	0.1 M KHCO_3_	- 0.7	1.2 (total)	(Xu et al., 2020)
Cu-N-C	**55% EtOH**	0.1 M CsHCO_3_	−1.2	16 (total)	(Karapinar et al., 2019)

**Doped/(halide-)modified copper catalysts**					

dodecanethiol-modified CuBr	72% C_2+_ (**35.9% EtOH**)	0.5 M KCl	−1.25	∼9 (EtOH)	(Wang et al., 2021)
P-doped Cu (P 8.3%) (Cu_0.92_P_0.08_)	64% C_2+_ (**EtOH 15%**)	1 M KOH	−0.7 to −0.75	210 (total)	(Kong et al., 2021)
Fluorine-modified Cu	85.5% C_2-4_ (**15% EtOH**, 65.2% C_2_H_4_)	1 M KOH	−0.89	800 (total)	(Ma et al., 2020b)
Boron-doped Cu	79% C_2_ (52% C_2_H_4,_**27% EtOH**)	0.1 M KHCO_3_	−1.1	10 (C_2_ products)	(Zhou et al., 2018)
Cu_2_S-Cu	**32% C**_**2+**_**-alcohols (25% EtOH, 7% PrOH)**	1 M KOH	−0.92	120 (C_2+_ alcohols)	(Zhuang et al., 2018)

**Copper alloys/copper-metal catalysts**					

CuPb-0.7/C (H-type cell)	73.5% C_2+_ (40.3% C_2_H_4,_**16.7% EtOH, 12.1% n-PrOH**, 4.4% AcOH)	0.1 M KHCO_3_	−1.3	–	(Wang et al., 2020a)
CuPb-0.7/C (GDE)	**29.9% EtOH** **1.43% n-PrOH** 16.3% AcOH	1 M KOH	> −1.5	400 (total)	(Wang et al., 2020a)
PdCu alloy foam (Pd_9_Cu_91_)	**13.7% n-PrOH**	0.5 M KHCO_3_	−0.65	∼1.2 (PrOH)	(Rahaman et al., 2020)
Ag_2_Cu_2_O_4_	**< 30% EtOH**	1 M CsHCO_3_	–	400 (total)	(Martić et al., 2020)
OD-Cu_90_Zn_10_ cubes	**20.2% EtOH** **2.1% PrOH** (33.6% C_2_H_4_)	0.1 M KHCO_3_	−1.1	–	(da Silva et al., 2020)
Multimetallic CuAgHg	**32% EtOH**	0.1 M KHCO_3_	−1.1	<10 (total)	(Kim et al., 2020b)
Au@Cu_2_O yolk-shell NPs on carbon lcoth	**52.3% EtOH**	0.1 M KHCO_3_	−0.3	<15 (total)	(Zhang et al., 2020a)
ZnO layer on top of Cu on carbon paper	78% C_2+_ (49% C_2_H_4_)	1 M KOH	−0.73	466 (C_2+_ products)	(Zhang et al., 2020b)
Ag_15_Cu_85_	**33.7% EtOH** **6.9% PrOH**	0.5 M KHCO_3_	−1.0 −0.9	8.7 (EtOH) 1.8 (PrOH)	(Dutta et al., 2020)
Cu-OD + Ag (20 nm)	**16.4% EtOH** 14.9% C_2_H_4_	0.1 M KHCO_3_	−1.1	4.1 (EtOH)	(Ting et al., 2020)
Ag-decorated Cu_2_O nanocubes	30% C_2+_liquids (**17% EtOH, 4% PrOH**)	0.1 M KHCO_3_	−1.0	<15 (total)	(Herzog et al., 2021)
Cu + Bibased MOFs	36.9% alcohols 8.6% MeOH **28.3% EtOH**	0.5 M KHCO_3_	−0.67	20 (total)	(Albo et al., 2019)
Ag_0.14_/Cu_0.86_	**41% EtOH**	1 M KOH	−0.67	250 (total)	(Li et al., 2019b)
ZnO/CuO	48.6% C_2+_	0.1 M KHCO_3_	−0.68	97 (C_2+_ products)	(Ren et al., 2019)
Cu-Ag	60% C_2_H_4_ **25% EtOH**	1 M KOH	−0.7	180 (C_2_H_4_)	(Hoang et al., 2018)

**Other catalysts**					

FeTTP[Cl] on Cu (sputtered on PTFE)	**41% EtOH**	1 M KHCO_3_	−0.82	124 (EtOH)	(Li et al., 2020)
Micropores in N-Doped mesoporous carbon	**78% EtOH**	0.1 M KHCO_3_	−0.56	∼0.2 (EtOH)	(Song et al., 2020)
Cobalt corrol complex on carbon paper	**48% EtOH**	0.1 M NaClO_4_	−0.56	2.5 (total)	(Gonglach et al., 2019)
Ag-Graphene-NCF	**79.1%–85.2% EtOH**	0.1 M KHCO_3_	−0.5 to −0.7	0.3 (total)	(Lv et al., 2018b)

Nevertheless, catalysts of various compositions already achieved FEs above 50% for multicarbon products. Best results were obtained with up to 85% FE_EtOH_ using Ag-graphene-NCF (Nano Carbon Fibers) as the catalyst, but the resulting current density was less than 1 mA cm^−2^, essentially not allowing any conclusive results on potential applications in larger scale (Lv et al., 2018b). Catalysts made of Cu-N-C (Karapinar et al., 2019), Cu-NPC (Han et al., 2020a), or consisting of micropores in N-doped mesoporous carbon (Song et al., 2020) also reached high Faraday efficiencies above 55% for ethanol. However, all of these catalysts/electrodes were operated at industrially irrelevant current densities of less than 20 mA cm^−2^. An intriguing question is what the performance or product distribution of these catalysts and electrodes will be at higher current densities. In contrast, higher current densities with simultaneously increased FEs for ethanol were obtained with Cu sputtered on PTFE and NC (FE_EtOH_ 52% at partial current densities of 156 mA cm^−2^) (Wang et al., 2020b) or N-doped graphene quantum dots on Cu-OD Cu nanorods with a FE_C2+ alcohol_ of 52.4% at a total of 282 mA cm^−2^ (Chen et al., 2020a).

As can also be seen from Table 1, the most frequently used electrolytes are KHCO_3_, CsHCO_3_, and KOH. However, because the influences on the resulting selectivity of the catalysts is multifactorial and involves not only the electrolyte but also other aspects such as cell design, membrane, temperature, and other parameters, the influence of those is discussed in detail in the chapter “Process Conditions”.

### Catalyst design

#### Catalyst syntheses

The syntheses of solid electrocatalysts, which are capable of producing ethanol during the electrochemical reduction of CO_2_, are manifold. In the most common cases, precipitation methods or electrodeposition were used, as well as sputtering of thin films. To further optimize the performance of the catalysts, surface modifications or reconstructions were also frequently carried out, or the catalyst layer was created by means of evaporation (e.g. via chemical vapor deposition).

##### Electrodeposition

**Figure 4:**
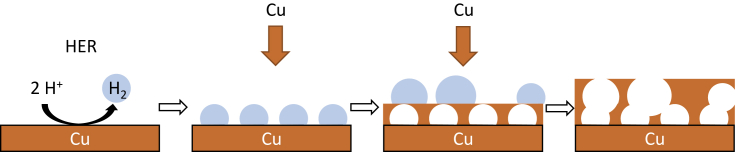
Schematic illustration of the electrodeposition of porous copper on a copper substrate with H2 bubbles as geometric template Own representation based on Dutta et al., 2016.

During electrodeposition, the catalyst is plated directly onto a substrate from an electrolyte solution, whereby the substrate is used as a working electrode and the deposition can be galvanostatic or potentiostatic. Electrodeposition has so far been used to coat gas diffusion layers, like carbon paper (Aeshala et al., 2012; Hoang et al., 2017, 2018; Lee et al., 2017; Kong et al., 2021), but also other substrates like metal foams, polished Cu discs, or Cu foil (Dutta et al., 2016, 2020; Ren et al., 2016; Rahaman et al., 2017, 2020; Kim et al., 2020b), which were then often applied in H-cells. Often, these catalyst materials were deposited from sulfuric acid, CuSO_4_, and other metal-sulfate-containing electrolytes (Aeshala et al., 2012; Dutta et al., 2016, 2020; Ren et al., 2016; Hoang et al., 2017, 2018; Rahaman et al., 2020). In addition, additives such as sodium citrate (Dutta et al., 2020) or citric acid (Kong et al., 2021), 3,5-diamino-1,2,4-triazole (DAT) (Hoang et al., 2017, 2018), as well as lactic acid (Ren et al., 2016; Lee et al., 2017) were added to the electrolyte solution. Sodium citrate was used in the deposition of Ag_15_Cu_85_-foam on Cu foil (Dutta et al., 2020). The deposition was realized from silver and copper(II)-sulfate-containing electrolyte at 3 A cm^−2^. In this process, the competing HER commonly results in the formation of gas bubbles as a geometric template for foam formation. Figure 4 schematically shows the process of deposition of porous copper using the resulting hydrogen as a template. The sodium citrate used should have an impact on the growth characteristics through chemisorption at the cathode surface. However, the electrodeposition of foams on Cu wafers was also successfully carried out without additives using sulfuric acid/CuSO_4_ solution at 3 mA cm^−2^ (Dutta et al., 2016). Owing to the mesoporous structure of the resulting Cu foam, there is an increased formation of C_2_ products such as ethane and ethylene. In the case of the Cu-Ag foams, subsequent calcination at 200°C and the associated formation of Cu_2_O also led to increased Faraday efficiencies for EtOH and PrOH of up to 33.7% and 6.9%, respectively (Dutta et al., 2020). Calcination was also carried out following the electrodeposition of Cu dendrites on electropolished meshs (Rahaman et al., 2017) and a Cu-Pd foam on Cu foils (Rahaman et al., 2020), to activate the catalyst as this thermal treatment may result in a higher FE for ethanol instead of CO due to segregation of the phases (Dutta et al., 2020). Zeng et al. electrodeposited Cu onto carbon paper and used thermal annealing to dope the Cu with phosphorus at 400°C and under N_2_ atmosphere using NaH_2_PO_2_∙H_2_O (Kong et al., 2021). The yield of C_2+_ products was thus increased by 1.9 times, and the FE for EtOH was even 2.8 times higher (15%) than without any doping. Another used additive is (3,5-diamino-1,2,4-triazole) DAT, which acts as an inhibitor for Cu deposition before reaching −0.18 V (vs RHE) (Hoang et al., 2017, 2018). As a result, it was possible to deposit Cu films with a high surface area and activity for CO_2_RR. It was possible to achieve 5–6 times higher current densities, when DAT was used as an additive in the deposition process than without (Hoang et al., 2018). Lactic acid was also used as an additive as it stabilizes the Cu ions in the solution (Ren et al., 2016; Lee et al., 2017).

##### Precipitation methods

Another commonly used method for catalyst synthesis is precipitation, and a broad variety of starting materials and products have been used or obtained. In some cases, the syntheses were carried out in the microwave, such as a precipitation reaction for Bi-MOFs (Albo et al., 2019), or in autoclaves as in the synthesis of Cu(OH)F from Cu(II) nitrate in DMF, with the addition of NH_4_HF_2_ for 4 h at 160°C (Ma et al., 2020b). In CO_2_RR, the resulting Cu(OH)F catalyst enabled the formation of C_2+_ products with FEs up to 65.2% at a maximum current density of 800 mA cm^−2^. Another example of the use of autoclaves is the preparation of paramelaconite (Cu_4_O_3_) from a Cu(II) nitrate-DMF-EtOH mixture after the addition of formic acid and dimethylamine at 130°C (Martić et al., 2019). The catalyst achieved an FE for C_2+_ products of over 61%. Cu nanoparticles can also be prepared by precipitation over the formation of copper oxides as shown by Jiao and coworkers. They precipitated Cu(OH)_2_ nanorods from a mixture of aqueous copper nitrate solution with ammonia and converted them to porous CuO by thermal annealing in the following (Lv et al., 2018a). After applicating the nanorods onto a GDL, the reduction to copper nanoparticles was performed at 10 mA cm^−2^. Another example is the precipitation of Cu(OH)_2_ followed by thermal annealing under a H_2_/Ar atmosphere. The catalyst was then partially oxidized by storing it in air before applying it to CO_2_RR (Shang et al., 2019). This procedure yielded core-shell Cu@Cu_2_O catalysts, which led to an FE of EtOH of 29% during CO_2_RR. Another example is the precipitation of Cu nanoparticles from a Cu(II)-containing solution with the addition of NaBH_4_ (Ma et al., 2016; Wei et al., 2020), which led to an FE for C_2+_ products of up to 80% (Wei et al., 2020). If a NaBH_4_ solution is combined with a CuCl_2_ solution, boron-doped copper can be obtained as a precipitate, which achieves FEs for EtOH up to 27% (Zhou et al., 2018). In addition to pure Cu precipitates, mixed oxides as well as other compounds with several metals have been successfully synthesized via precipitation and used for electrochemical CO_2_ reduction. For example, a catalyst of graphene oxide, ZnO, and Cu_2_O was prepared by precipitation and produced up to 30% propanol (Geioushy et al., 2017). Precipitation of Ag_2_Cu_2_O_3_ with aqueous NaOH from a solution containing Cu and Ag nitrate was also successfully carried out under inert conditions, and the Faraday efficiency for this catalyst was about 25% for EtOH (Martić et al., 2020). Another catalyst that produced nearly 30% ethanol, when used in a GDE, is a CuPb-0.7/C (Pb shell thickness is 0.7 nm) catalyst, which was precipitated from a copper acetate, PbCl_2_, ascorbic acid, diphenyl ether, and oleylamine-containing solution (Wang et al., 2020a). With 25% FE, slightly less ethanol was produced by V-Cu_2_S nanoparticles, which were prepared using Cu acetylacetone and dodecanethiol (Zhuang et al., 2018).

##### Sputtering

Another method for the preparation of catalysts or electrodes, which has already led to materials providing high Faraday efficiencies at industrially relevant current densities, is sputtering. In most cases, Cu was sputtered from a pure Cu target onto a PTFE membrane (Dinh et al., 2018a; Gabardo et al., 2019; García de Arquer et al., 2020; Li et al., 2020) (pore size 0.45 μm). Subsequently, either carbon black (Dinh et al., 2018a; Gabardo et al., 2019), graphite (Gabardo et al., 2019), Nafion, or a mixture of Nafion and Cu-NPs (García de Arquer et al., 2020) was spray coated onto the sputtered layer. Spray coating of porphyrin-based complexes (FeTTP) onto sputtered copper ultimately resulted in an FE for ethanol of 41% (Li et al., 2020), as did co-sputtering of Cu and Ag onto a PTFE membrane (Li et al., 2019b). The highest EtOH yield was obtained with an FE of 52% by first sputtering Cu and then a layer of N-C onto the membrane (Wang et al., 2020b).

##### Evaporation methods

Some catalysts were also synthesized via evaporation and vapor deposition onto a substrate. For example, compared to pure Cu, alcohol formation occurred at >265 mV more positive electrode potentials on a polycrystalline Cu foil coated with gold (Carlos G. Morales-Guio et al., 2018). Furthermore, CVD of boron- and nitrogen-doped diamond on a Si substrate was performed and the resulting electrode led during CO_2_RR to an FE of 93.2% for ethanol, but with current densities below 2 mA cm^−2^ (Liu et al., 2017).

##### Modification/reconstruction of surfaces

For modifying or reconstructing the surface of Cu foils/substrates, various ways including electrochemical and plasma activation were used. However, the resulting catalysts were always used in H-type cells, which lead to very low current densities. One possibility of surface modification for copper foil is to cyclize it. For example, the FE for ethanol could be increased from 2.2% to 7.7% by cyclizing the foil for three cycles between −1.1 and 0.9 V for 20 mV/s in a 0.1 M KHCO_3_ solution, containing 4 mM KCl (Schouten et al., 2011). Cyclization in copper nitrate solution led to the formation of single crystal Cu_2_O nanocubes and an FE for C_2+_ products of 60% was obtained (Jiang et al., 2018). Another possibility to modify the catalysts surfaces is plasma activation in O_2_ plasma (FE_C2+_ 69%) (Gao et al., 2018) or heating a copper substrate in an oven to 1100°C followed by quenching in air, leading to the formation of sponge-like structures and an FE for C_2+_ products of 70% (Lei et al., 2020). Wet chemical modification of the surface by oxidation with H_2_O_2_ and diluted HCl leads to the formation of CuCl on the surface, followed by the formation of Cu_2_O by immersion in KHCO_3_ (Kibria et al., 2018). Subsequent electrochemical CO_2_ reduction then led to FEs for C_2+_ products above 80%. Also, modification with halides was obtained by immersing Cu foils in solutions containing CuBr_2_ (Wang et al., 2021). Here, CuBr tetrahedrons formed on the surface which were subsequently immersed and thus uniformly coated in dodecanethiol. The application of the coated catalyst in CO_2_RR resulted in almost 36% FE for EtOH.

##### Other methods & applicability assessment

Apart from the synthesis routes described so far, various catalyst syntheses can be found which were only used by a few groups including special synthesis routes—e.g. a 4-step organometallic synthesis of co-corroles (48% EtOH, −0.56 V, total 2.5 mA cm^−2^) (Gonglach et al., 2019), the synthesis of Cu-N-C by low-energy ball milling followed by pyrolysis in an argon stream (55% EtOH, −1.2 V, total 16 mA cm^−2^) (Karapinar et al., 2019), the impregnation of melamine foam in a silver nitrate-graphene oxide solution followed by calcination (79%–85% EtOH, −0.5 to −0.7 V, total 0.3 mA cm^−2^) (Lv et al., 2018b), or the preparation of carbon supported Cu catalysts by using an amalgamated Cu-Li method (91% EtOH. −0.7 V, total 1.2 mA cm^−2^) (Xu et al., 2020).

Regarding the synthesis and study of electrocatalysts versus industrial applicability, our group has recently published a perspective article (Siegmund et al., 2021). There we defined the following evaluation criteria: (1) The issue of complexity and price required to synthesize the catalyst: Synthesis routes such as multiple steps synthesis are considered problematic in this regard, as they are accompanied by great complexity, as well as costly purification steps. Precipitation reactions, sputtering, or electrodeposition, on the other hand, are in simple principle and can be carried out in just a few steps. The processes described under “Surface modification” can also be described as predominantly less complex. (2) The issue of producing the catalyst in sufficiently large quantities (Siegmund et al., 2021): E.g. it is possible to sputter large areas without any problems, which is already used for the production of thin-film solar cells (Edoff, 2012). Precipitation reactions are also common processes in industry and have the potential to be carried out on a large scale, as does electrodeposition of metals. However, individual considerations would need to be given to each catalyst synthesis in terms of its scalability. More problematic are synthesis routes which contain discontinuous processes, e. g. evaporation processes. (3) The issue of (long-term) stability of the catalyst materials at relevant current densities (Siegmund et al., 2021): Some catalyst materials mentioned above have already been tested for their stability over longer time periods, e.g. Co-corroles showed stable electrolysis over 140 h, but at very low current densities of −2.5 mA cm^−2^ (Gonglach et al., 2019). Also, sputtered electrodes were already stable over 150 h electrolysis (at up to 100 mA cm^−2^) (Dinh et al., 2018a). For many of the catalysts, however, evidence of long-term stability under industrially relevant conditions is lacking, which is urgently needed to evaluate the applicability of the materials.

#### Structural properties and crystal orientations

In addition to the composition of the catalyst, its surface morphology and crystal face orientation were determined to be decisive factors in the selective reduction of CO_2_ to C_2+_ alcohols and therefore the factors that increase the FE for multicarbon product formation are discussed here.

Several studies have already shown that Cu(100) surfaces are more selective for C_2+_ products, while Cu(111) is more likely to lead to the production of CH_4_ (Jiang et al., 2018; Wang et al., 2019; Gregorio et al., 2020; Han et al., 2020b; Ting et al., 2020). However, an excess of CO at Cu(111) sites could also lead to EtOH formation. Cu(100), on the other hand, supports the dimerization of ∗CO, which is formed as intermediate (Han et al., 2020b). The selectivity via the surface orientation is also evident when using Cu nanocubes and Cu nanospheres. As more Cu(100) is present on the surface in the former, the ethylene formation under alkaline condition is more pronounced (Jiang et al., 2018; Wang et al., 2019). Another example for the advanced C-C coupling on Cu(100) can be observed on CuCl-derived Cu electrodes as they show an increased selectivity for C_2_ products (Kibria et al., 2018). Compared to electropolished electrodes, those CuCl-derived ones show a change in preferential crystal orientation from Cu(111) to Cu(100). Upon transition from Cu(111) to Cu(100), FEs for C_2+_ products increased from 30% to 73%, that of propanol from 0% to 5%.

**Figure 5:**
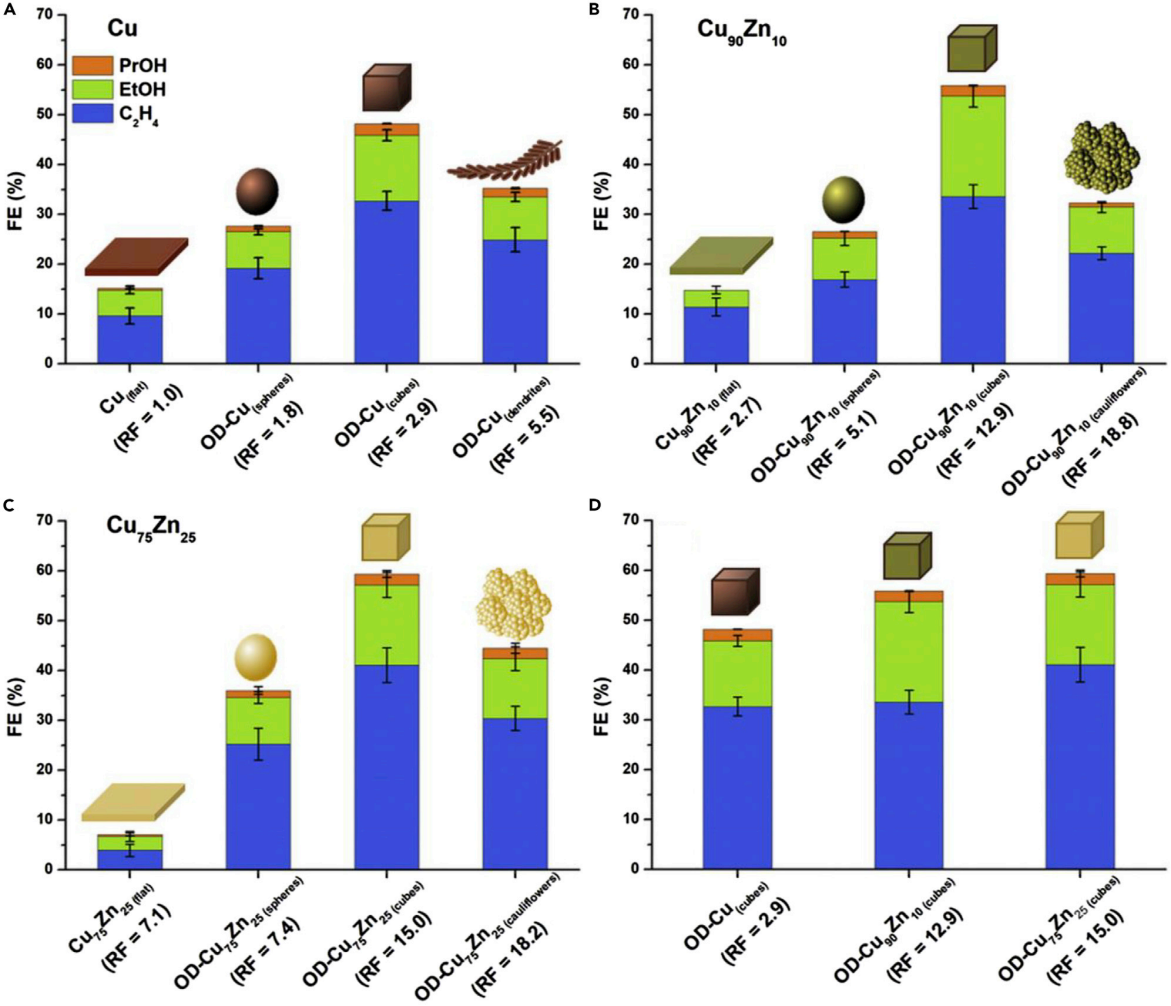
Example of the impact of catalyst morphology and composition for catalyst systems based on Cu or Cu/Zn on the Faraday efficiencies of C_2+_ products at −1.1 V with simultaneous indication of the roughness factor (RF) (A) Influence of the morphology of pure Cu catalysts. (B) Influence of the morphology of Cu_90_Zn_10_ catalysts. (C) Influence of the morphology of Cu_75_Zn_25_ catalysts. (D) Influence of the Cu:Zn ratio for cubic catalysts. Adapted from *Journal of Electroanalytical Chemistry* (da Silva et al., 2020), applying terms of CC BY licens.

When comparing Cu cubes and Cu octahedrons, the formation of C_2_H_4_ was also highest at the cubes, whereas CH_4_ formation was more pronounced at the octahedrons (Gregorio et al., 2020). Furthermore, it was shown, using Cu-Zn catalysts as an example, that the roughness factor of the surface directly influences the product distribution. Higher roughness correlated with higher FEs for C_2+_ products (da Silva et al., 2020). Figure 5 shows the influence of surface morphology on CO_2_RR in terms of C_2+_ product distribution and the influence of Cu-Zn ratio on catalytic activity. While the Faraday efficiency for the formation of C_2+_ products increases with increasing surface roughness, it simultaneously decreases for CH_4_ and H_2_ (Jeong et al., 2020). The presence of corners and steps on the surface promotes the adsorption of C_1_ products and this, in turn, leads to an improvement in the dimerization to C_2+_ products (Hoang et al., 2018). The improvement in C_2+_ production due to both more sharply defined structures and more curved surfaces is expected to occur as a result of improved bubble nucleation, a concentration of stabilizing cations as well as high local fields and thus increased current density (Luna et al., 2018). Electro-redeposition is expected to lead to these electronic and morphological effects, which improves selectivity and activity of Cu in the production of C_2+_ during CO_2_RR. Furthermore, the yield of C_2_-C_3_ products could be significantly increased by *in situ* structural transformation of densely packed Cu-NPs by electrolysis to cube-shaped catalytically active structures (Kim et al., 2017).

Besides the surface roughness, porosity also plays an important role in the electrochemical performance of CO_2_RR (Han et al., 2020a). For example, the transport of CO_2_ through the electrolyte-electrode interface at high current densities is facilitated when using GDEs with highly porous structures (Lv et al., 2018a). In addition, the micropores are also expected to play an important role in the adsorption capacity of CO_2_ by the catalysts (Han et al., 2020a).

In addition to the catalysts themselves, the type of electrode and its manufacture also have a significant influence on the final performance in CO_2_RR (Tan et al., 2020). Catalyst ink-based preparation techniques, for example, offer the possibility to influence catalyst surfaces via multiple parameters. In addition to using different techniques such as dropcasting, airbrushing, or hand painting, the drying temperature can also be adjusted. Overall, thinner porous catalyst layers, e.g. obtained by dropcasting or hand painting, should result in fewer C_2+_ products being formed. If, on the other hand, the catalyst layer is enlarged, there is better CO_2_ mass transfer within the porous layer. Simulations suggest that the layer thickness is more important than the porosity for controlling the local concentration of CO_2_ (Tan et al., 2020). In addition to the layer thickness, the loading of catalyst also influences the results. For example, the study of Cu-NPs in combination with pyridinic N species in N-doped porous carbon showed that a copper loading of 10% was not sufficient, whereas 30% was too much and led to preferential ethylene formation instead of EtOH and PrOH. The highest yields for multicarbon alcohols were obtained at 20% Cu loading (Han et al., 2020a).

#### Copper-oxide & oxide-derived (OD) copper catalysts

Whether to use copper, copper oxide, or OD-copper electrodes is a frequently discussed topic. In comparison to pure copper electrodes, oxide-derived copper electrodes contain remaining oxides, which should simplify the adsorption of ∗CO and the C-C coupling (Ting et al., 2020). Thus, OD-Cu should increase the selectivity for C_2+_ products (Iijima et al., 2019). Furthermore, investigations have shown that a thin layer of metastable Cu_2_O on an electrode made of OD-Cu can result in an increase in selectivity in favor of C_2_ products due to an improved stabilization of intermediates of CO_2_RR (Shah et al., 2020). It was also shown that current densities are higher on plasma-activated Cu foil (CuO_2_) than on electropolished Cu (Singh et al., 2016; Gao et al., 2018). This is not due to structural changes but can rather be understood as a chemical effect of Cu^+^ species. An increase in the product ratio for CO_2_RR of C_2+_/C_1_ with FEs of up to 61% for C_2+_ products was also shown by using a GDE, which is carbon-based and contains Cu derived from Cu_4_O_3_. Partial current densities of 185 mA cm^−2^ were obtained and due to the same reaction paths and intermediates of EtOH and ethylene, an increase in both C_2_H_4_ and EtOH yield was obtained by improvement with OD-Cu compared to normal Cu (Martić et al., 2019). An improvement in C_2+_ selectivity was also recently achieved by using Cu catalysts with nanocavities in which carbonaceous intermediates are trapped (Yang et al., 2020). The intermediates would not only cover the surface of the catalyst but also stabilize the Cu^+^ present there, which is thus also retained during CO_2_RR and allows the selectivity to be increased (75.2% FE at 267 mA cm^−2^) (Yang et al., 2020).

However, other investigations show that only metallic copper is active, while oxides remaining in OD electrodes are unstable and inactive under CO_2_RR conditions during catalysis (Ting et al., 2020). Spectroscopic investigations have shown that there is a low CO intermediate formation on Cu_2_O, resulting in a low activity toward CO_2_RR. According to Han and coworkers, CO_2_RR takes place on Cu^0^ and not Cu^+^ or Cu^2+^ and the oxides are not decisive for selectivity toward C_2+_ products. Instead, they examined the grain sizes and found a decrease of selectivity in the order Cu^0^ > Cu^+^ > Cu^2+^, and that the reduction of the oxides leads to fragmentation and thus to an increase in surface roughness (Lei et al., 2020). A direct comparison of electropolished Cu electrodes with those containing Cu oxide or Cu hydroxide showed that electrodes containing Cu oxides or hydroxide showed better selectivity for C_2+_ products while suppressing the formation of CH_4_ (Lei et al., 2020). The best results were obtained with Cu oxide electrodes with an FE of 68.2% and up to 64 times higher current densities than the pure Cu electrode. In the catalyst, three Cu species coexisted in different layers—Cu^0^, Cu^+^, and Cu^2+^. Within 1 h of CO_2_RR, all species were reduced to Cu^0^, but fragmentation to irregular nanoparticles also took place. The resulting network shows an enrichment of highly active sites, which facilitates CO adsorption. Furthermore, more high-index facets were exposed. These effects resulted in the improved selectivity (Lei et al., 2020). An investigation on Cu(100) surfaces using pulsed potential sequences (0.6 V and −1.0 V for 1 s each) also led to an increase in selectivity for C_2+_ products. While potentiometric measurements at −1 V on Cu single-crystal electrodes achieved FEs for EtOH of 8% and for ethylene of 45%, the overall value increased to 76% for the products, with ethanol FEs around 30%. The increased selectivity for ethanol is explained via a continuous *in situ* regeneration of Cu(I) and thus the co-existence of Cu(I) present as Cu_2_O and Cu(0) on the surface, the Cu(100) domain, and the defect sites (Arán-Ais et al., 2020).

Sargent and coworkers showed with the help of XAS measurements of GDEs that a direct reduction to metallic copper in the catalyst layer was achieved within 16 s, which implies that Cu^0^ is responsible for the selectivity toward EtOH and not the presence of oxides (Wang et al., 2020b). In addition to the question to what extent oxides themselves have an influence on the selectivity of CO_2_RR at Cu electrodes, the influence of interparticle distances between CuO_x_ nanoparticles was also investigated (Jeong et al., 2020). It was shown that increasing the distance between those NPs improves the C_2+_ selectivity, as long as it is still < 1nm. The C_1_ product formation was lowered and the obtained current densities were up to 12 times higher than with the unmodified catalyst. The reason for this was a higher surface roughness (increased ECSA) and a lowered energy barrier for CO_2_RR. Again, Cu^+^ was reduced to Cu^0^ during the reduction reaction (Jeong et al., 2020).

Another strategy is the combination of copper oxides with copper in the catalyst via the formation of Cu@Cu_2_O core-shell catalysts (Shang et al., 2019). The synergy between Cu^0^ and Cu^+^ leads to an increase in selectivity and efficiency in the formation of C_2+_ products, whereby dimerization should be facilitated by promoting the formation of a positive- and a negative-charged carbon atom (Shang et al., 2019).

#### (Surface) modifications with halides and organics

**Figure 6:**
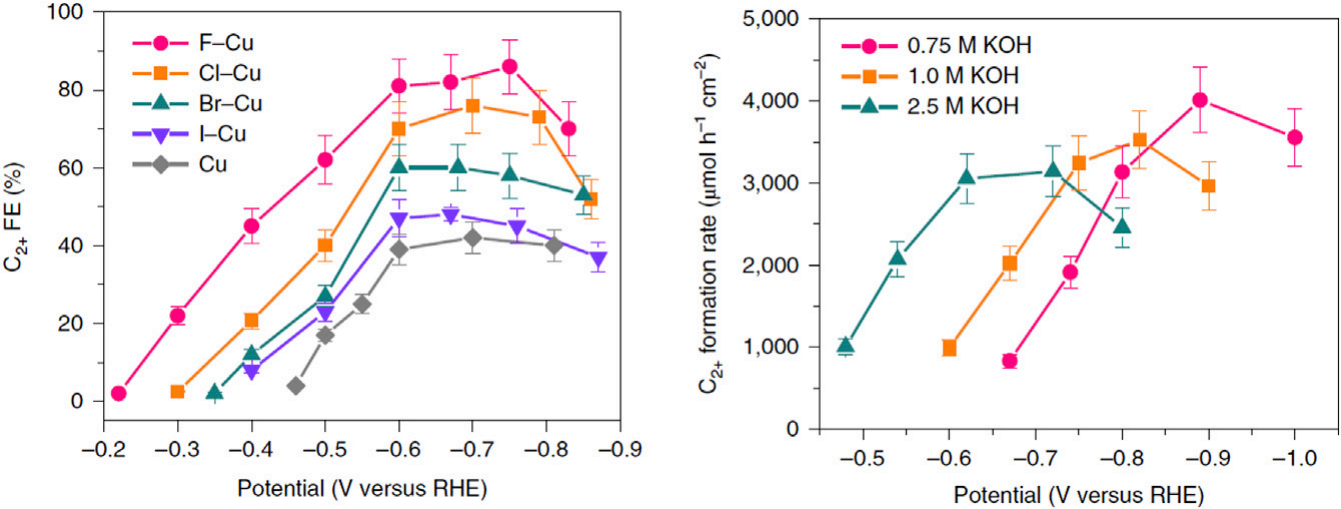
Influencing factors on FE_C2+_ product formation using Cu-halide catalysts (Left) Influence of halide type and potential in 1 M KOH (Right) Influence of KOH concentration and potential using a Cu-F catalyst. Reprinted by permission from *Nature Catalysis* (Ma et al., 2020b).

Besides the influence of surface activation or, for example, the use of OD-Cu electrodes, the influence of halides in Cu-based electrodes was also of interest for the CO_2_RR. Therefore, Wang and coworkers produced halide-containing copper catalysts via a precipitation process and found during electrochemical measurements in a flow cell that the adsorption capacity increases in the following order: Cu < I-Cu < Br-Cu < Cl-Cu < F-Cu (Ma et al., 2020b). Overall, the C-C coupling works better the higher the coverage of the surface with ∗CO is. The authors suggested that the presence of Cu^+^ sites may increase CO adsorption. In connection with C_2_H_4_ formation, they found that in the Cu-halides catalysts with increasing electronegativity of the halide, only a slight decrease of the onset potential could be observed. This indicates that the copper catalysts modification with halides promotes the first step after the ∗CO intermediate formation. Furthermore, a dependence on the local pH value was observed. Thus, a significant increase in C_2+_ formation (with FEs of EtOH up to 15%) for F-Cu catalysts with increasing local pH was observed when using different 0.5 M electrolytes in the following order: K_2_HPO_4_ < K_2_CO_3_ < K_2_SO_4_ (Ma et al., 2020b). Figure 6 shows both the influence of the halide on the formation of C_2+_ products and the influence of the KOH concentration for the Cu-F catalyst.

Another recently published study shows the production of a halide-containing copper catalyst by oxidative-reductive recycling of polycrystalline copper in KHCO_3_ solution with addition of the corresponding potassium salt (Han et al., 2020b). While Cl^−^ and Br^−^ stabilized Cu^+^ and thus tend to be promoters of Cu dissolution, I^−^ inhibited it by forming an almost insoluble polyhedral CuI, along with the associated passivation of the surface. This cycling of copper in KHCO_3_ solution resulted in different structures on the surface of the Cu electrode depending on the halide. Although the reconstructed (re) Cu-I electrode had less Cu(100) on the surface compared to re-Cu-Br and re-Cu-Cl electrodes, the best selectivity for these electrodes was obtained for the copper electrodes modified with iodide with 80% FE_C2_. XAS measurements showed the same ratio of Cu^0^ to Cu^+^ for all three electrodes, rendering it not decisive for the selectivity. However, a correlation of the electrochemical performance during CO_2_RR was observed with the porous, in the case of re-Cu-I intertwined and spiderweb-like, hierarchical structure on the surface. The intermediately generated CO is supposed to be trapped inside the pores, providing an increased ∗CO-coverage, which leads to an increased dimerization (Han et al., 2020b). The question of how the addition of halides in the electrolytes affects the electrochemical performance of the catalysts and electrodes is discussed in the following chapter about process conditions.

In addition to the previously discussed modifications of the catalysts with halides, there are also investigations on the influence of hydroxide. It was shown that the presence of OH groups near the catalyst surface improves the reaction kinetics and stabilizes the oxygen in Cu_x_O catalysts during the reduction reaction (Xiang et al., 2019). Also, with increasing number of OH^−^ bound to Cu, the adsorption of CO and thus also dimerization should be supported (Iijima et al., 2019). The adsorption energy of CO will be increased compared to pure Cu surfaces, because the OH layer will probably bring CO molecules closer together while a simultaneous reduction to C_2+_ products takes place.

A further modification reported in the literature is the coating of a Cu foil with a 50 nm thick polyaniline film (PANI), whereby an improvement of the C_2+_ selectivity from an FE from 15% to 60%, for the coating of Cu nanoparticles even to 80%, was achieved (Wei et al., 2020). The PANI layer is intended to increase the coverage of the surface with CO and improves the interaction of these molecules. At the same time, HER is significantly reduced, probably due to the increased hydrophobicity. Moreover, Mougel and coworkers created a superhydrophobic surface on their applied electrode by treating Cu dendrites with 1-octadecanthiol, resulting in an FE of 56% for C_2_H_4_ and 17% for EtOH at neutral pH (Wakerley et al., 2019). The gas was captured at the electrode-electrolyte interface, which resulted in an increase in CO_2_RR and C_2+_ selectivity. Modification of surface hydrophobicity and adsorption energies is also possible by combining the use of halides and organic compounds (here dodecanethiol) (Wang et al., 2021). Dodecanethiol lowers the selectivity for H_2_ and CH_4_ by decreasing the amount of adsorbed H∗. The bromide introduced into the copper catalyst, on the other hand, shifts the selectivity to ethanol by stabilizing positive Cu valence sites, which are expected to have a significant effect on the product distribution in CO_2_RR (Wang et al., 2021).

#### Bifunctional catalysts and copper alloys

**Figure 7:**
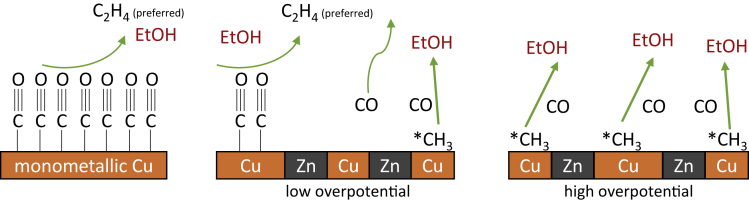
Suggested mechanism for the formation of ethanol at Cu-Zn catalysts at different potentials Own representation based on Ren et al., 2019.

In addition to varying the oxidation states of copper or creating specific structures on the catalyst surface, bifunctional catalysts can be used to improve the selectivity for C_2+_ products. The potential for the formation of CO at the co-catalyst should correspond to the potential range for the formation of the target product at copper (Ren et al., 2019). As discussed before in multimetallic and bifunctional catalysts, the combination with ZnO can increase the C-C coupling kinetics by increasing the local concentration of the intermediate CO (Zhang et al., 2020b). In the case of Cu/ZnO tandem electrodes, additional CO was generated at the ZnO, and the resulting CO excess increased the C_2+_ selectivity by facilitating C-C coupling. The electrodes showed a stability of 10 h at 600 mA cm^−2^ (Zhang et al., 2020b). The use of ZnO for increased selectivity of C_2+_ products in a Cu/ZnO tandem catalyst as a bifunctional catalyst with different domains was also shown by other groups. Grätzel and coworkers modified CuO nanowires via atomic layer deposition with ZnO, thus shifting the selectivity of CO and HCOO^−^ (selectively formed on Cu nanowires) toward EtOH (Ren et al., 2019). Herein, the additional active sites of zinc available for CO intermediate formation increase the amount of CO for C-C coupling and thus reduce HER at the same time. Figure 7 shows the proposed mechanism in more detail including the impact of varying the overpotential. Higher overpotentials lead to higher production of ∗CH_3_, which can then be coupled with CO to form ethanol. Another example for bifunctional catalysts in CO_2_RR is the combination of copper with silver for obtaining enhanced yields for C_2+_ product (Hoang et al., 2018). Sargent and coworkers made efforts in designing catalysts that favor the CO_2_RR pathway to ethanol. The diverse binding sites, existing in Ag-Cu bimetallic catalysts, led to a destabilization of the ethylene intermediates, probably due to a disruptive influence of Ag on ethylene-forming Cu sites. This resulted in an increased ethanol selectivity of 41% at −0.67 V vs. RHE, compared with an FE of 29% at best for the pure Cu catalyst (Li et al., 2019b). Cu-Pd foams also revealed good catalytic activity toward CO_2_RR to C_2+_ products. This catalyst shows phase segregation in the nm range, with Cu- and Pd-rich domains present. These lead to a 2 times higher selectivity toward PrOH instead of EtOH. The methane pathway (C_1_) is suppressed and a concerted spillover effect of ∗CO and ∗H adsorbed on Pd domains results in the preferential formation of C_3_ products (Rahaman et al., 2020). A catalyst for selective alcohol formation is an OD-Ag-Cu-foam of stoichiometry Ag_15_Cu_85_ (Dutta et al., 2020). CO is selectively formed in the silver domains and is transferred by surface diffusion to copper, where it is converted to alcohols by C-C coupling. The excess of CO at the catalysts surface leads to good selectivity with up to 34% FE for ethanol. Furthermore, a selective activation of the copper by oxide deposition and the subsequent reduction under CO_2_RR conditions takes place and enhances the selectivity as well. Doping biphasic (BP) copper(I) oxide with silver also yields significant improvements in EtOH yield, including a shift in product selectivity from ethylene to divalent alcohols (Lee et al., 2017). The FE for EtOH was raised from 11% to 35% for Ag-Cu_2_O_BP_ compared to the undoped catalyst. Another option is the destabilization of the ethylene reaction path in favor of an increased EtOH production by using a Ag/Cu-alloy phase catalyst (Li et al., 2019b). Ethylene is preferentially formed at highly coordinated surfaces and the introduction of an element with a weaker bonding capacity to carbon than copper reduces the probability of the formation of ethylene intermediates by increasing the variety of available bonding sites. On Cu (111), there are four bonding sites available, on Ag-doped Cu (111), there are 16.

A concept that has been applied several times is the use of core-shell catalysts, where CO is enriched inside the nanocaves by reduction of the core, and is then converted by the shell into the target product (Zhuang et al., 2018; Ren et al., 2019; Shang et al., 2019; Zhang et al., 2020a). An example for the production of ethanol is a catalyst consisting of Cu_2_O nanocavities with embedded gold nanoparticles, which shifts the selectivity for CO_2_RR from C_1_ to C_2_ products (Zhang et al., 2020a). The gold core reduces CO_2_ to CO in the nanocavities, resulting in a high local concentration of this intermediate. EtOH is then formed at the copper shell. Another core-shell catalyst developed by Sargent and coworkers consists of a Cu_2_S core and a Cu-V shell (Zhuang et al., 2018). This catalyst achieved an FE of 32% for alcohols, with 25% for EtOH and 7% for PrOH at a partial current density of 120 mA cm^−2^, resulting in a 6-fold improvement of the EtOH: ethylene ratio from 0.18 to 1.2 compared to pure Cu nanoparticles.

Another possibility for increasing selectivity toward multicarbon alcohols is the use of metal organic frameworks. The use of Cu(II)- and Bi(III)-based MOFs resulted in a FE_EtOH_ of 28.3% (Albo et al., 2019). However, these electrodes are only stable for 5 h. The increased EtOH formation can be explained by the reduction of CO_2_ at Bito HCOO^−^, which is then transferred to Cu and reduced to alcohol. Owing to longer diffusion paths within the MOFs compared to other catalysts, a longer contact of the products is guaranteed and a reduction of MeOH to EtOH under C-C coupling can take place. A longer stability with up to 140 h was achieved for electrodes by using Co-corrole carbon paper electrodes (Gonglach et al., 2019). The mechanism here is not based on CO as an intermediate but the formic acid pathway and Co-corroles stabilize various radical intermediates. EtOH could be obtained with an FE of 48%.

Furthermore, metals were incorporated into various carbonaceous support materials, e.g. an N-doped porous carbon-supported copper catalyst was used for CO_2_RR to multicarbon alcohols (Han et al., 2020a). Pyridinic N-species were probably the CO-producing sites and copper the catalytic sites for the production of EtOH and PrOH. An increase in pyridinic nitrogen atoms improved both selectivity and activity toward multicarbon alcohols. The carbon support influenced the copper concerning structure and size, resulting in improved CO_2_ adsorption and CO production. Pyridine nitrogen was also used in a catalyst consisting of Ag nanoparticles in a 3D-graphene-wrapped nitrogen-doped carbon foam, as it can bind ∗CO intermediates better than other N-species (Lv et al., 2018b). EtOH is then gradually formed at the Ag-NPs. The catalyst is also characterized by high conductivity. The direct comparison of Cu nanorods with nitrogen-doped graphene quantumdots (NGQ) and Cu nanorods clearly shows higher EtOH and PrOH yields (Chen et al., 2020a). The reason for the increased formation of multicarbon alcohols is the better stabilization of the oxygen-containing intermediates. As mentioned and discussed before in multimetallic and bifunctional catalysts, there is also a synergistic effect, as C-C couplings occur at both the copper nanorods and the NGQ, and the formation of the desired C_2+_ products is greatly enhanced by these dual active sites.

Besides the mentioned combination of metals and carbonaceous supports and the usage of bimetallic catalysts, a molecule-metal composite has been proposed. The porphyrin-based co-catalyst increased ∗CO coverage on the metal surface, promoting C-C coupling and favoring the ethanol pathway. The FE for ethanol was 41% at −0.82 V vs. RHE, higher than 29% FE at –0.84 V observed for pure Cu (Li et al., 2020). Molecular cobalt corrole complexes have been described, with the electron-donating ligands favoring a square-planar cobalt(I) complex as active species. It could reach an ethanol FE of 48% at −0.8 V vs. RHE (Gonglach et al., 2019). Also, acetate as potential C_2_ product could be obtained using a manganese corrole complex with 63% FE at −0.67 V vs. RHE (Schoefberger et al., 2020).

Metal-free catalysts have also already been used for selective EtOH production, e.g. N-doped mesoporous carbon. High local electrical potentials within the mesoporous channel walls lead to an improved activation of CO_2_. In addition, this also facilitates C-C coupling through the pyridine and pyrollic nitrogen atoms. The micropores contained in the channel walls increase the selectivity of the catalyst for EtOH as well as the reactivity (Song et al., 2020).

In recent years, there has been a steady stream of new investigations of CO_2_RR with constantly new catalysts (Table 1) and a wide variety of production methods (Chapter 3.2). Overall, although the catalyst has a great impact on the selectivity and efficiency of the electrosynthesis, it is very difficult to compare catalysts due to large differences in electrode preparation, the test setup itself, and different electrolyte solutions, pressures, temperatures, etc. Here, standardized cells and reaction conditions could help to classify the potential of the catalysts in a reasonable way. In this area, there are already initiatives such as NFDI4Cat, which deals with the sharing of metadata in the entire field of catalysis and thus aims to create a research data infrastructure (Wulf et al., 2021). Regarding a potential industrial application, an additional focus should be on simplicity, scalability, and the lowest possible cost of production, as well as on long-term stability as numerous catalysts have been tested only for their capability in reducing CO_2_ for few minutes. In addition, more emphasis should be given to the use of flow cells or MEAs for testing the catalysts to achieve higher current densities. Finally, as already discussed mechanistic studies, for example, by using *in situ* methods and carrying out of operando studies, should be given greater emphasis.

## Process conditions

Important for the successful electrolysis of CO_2_ to valuable products is not only the choice of the appropriate catalyst but also suitable process conditions.

### Electrolyte

One key parameter with a strong influence on catalyst/electrode performance is the electrolyte. For example, compared to KHCO_3_, a higher selectivity to carbonaceous products using KOH was shown. High local pH values, which can be favored by an electrolyte with low buffer capacity, have been shown to improve the product distribution toward higher hydrocarbons (Hori et al., 1989, 1997; Schouten et al., 2014; Varela et al., 2016; Xiao et al., 2016; Wang et al., 2018). Thus, alkaline electrolytes have been used in flow cells with promising results (Ma et al., 2016; Dinh et al., 2018a; García de Arquer et al., 2020).

#### General considerations – KOH vs KHCO_3_

**Figure 8:**
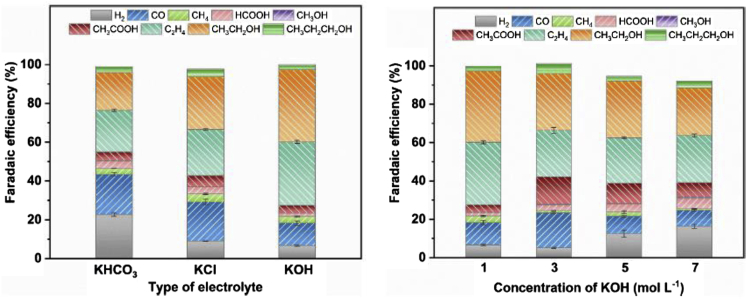
FEs using a poly(ionic liquid)-based Cu^0^-Cu^I^ tandem catalyst for CO_2_RR varying the electrolyte and the concentration of KOH electrolyte; 400 mA cm^−2^ Copyright Wiley-VCH. Reproduced with permission (Duan et al., 2021).

Owing to the competition between CO_2_ reduction and hydrogen evolution, alkaline conditions are required for an efficient performance of CO_2_ electrolysis (Pătru et al., 2019). In addition, the electrolyte used should be as conductive as possible in order to achieve higher energy efficiencies for the CO_2_RR (Dinh et al., 2018a). How much this affects the overall cell performance is shown by a comparison between 10 M KOH and 0.1 M KHCO_3_, according to which the ohmic losses in the formation of C_2_H_4_ were reduced by a factor of 47 under the highly alkaline conditions (Dinh et al., 2018a). Even when comparing 1 M KOH with 1 M or 0.1 M KHCO_3_, clear differences can already be seen. Although the same current densities can be achieved in principle with both electrolytes, the same current densities can be reached with 1 M KOH at considerably lower voltages, because the CO_2_RR activity is significantly higher there (Dinh et al., 2018b) — in a catholyte with a higher basicity, less energy is therefore required for the CO_2_RR (Xiang et al., 2019). In addition, the use of 1 M KOH also shifts the selectivity toward carbonaceous products (Dinh et al., 2018b; Lv et al., 2018a; Xiang et al., 2019). Thus, by changing from 1 M KHCO_3_ to 1 M KOH at an Ag/PTFE-GDE, instead of 80%, an FE of 90% for CO could be achieved (Dinh et al., 2018b). Furthermore, C_2_ products should be obtained mainly at KOH concentrations above 0.5 M (Xiang et al., 2019). An increase in FE for these was observed with a) more negative potentials and b) higher KOH concentrations. The current density was also significantly increased by a higher KOH concentration. Furthermore, OH groups in the vicinity of the catalyst surface should improve the reaction kinetics and, in the case of Cu_x_O catalysts, stabilize the oxygen of the catalyst during the reduction reaction (Xiang et al., 2019). However, a recent study by Zhang and coworkers showed the opposite trend with a decrease in overall C_2+_ product formation (from 76.1%, 1 M KOH) and ethanol with increasing KOH concentration 7 M (60.4%, 7 M KOH) (Figure 8 (right)) (Duan et al., 2021). The authors explained this deviation from previous publications with the high carbonate formation due to the high current densities of 400 mA cm^−2^ used. The described dependence on KOH concentration was performed on a poly(ionic liquid)-based Cu^0^-Cu^I^ tandem catalyst and also shows a significant increase of C_2+_ products for using 1 M KOH instead of 1 M KHCO_3_ or 1 M KCl. While the formation of hydrogen decreases from 22.7% (KHCO_3_) to 6.6% (KOH), the FE for ethanol increases significantly (Figure 8 (left) (Duan et al., 2021)). However, there are also studies which do not only deal with the basicity and thus the OH^−^ concentration, but focus on the cation of the electrolyte solution. Thus, there are also results that indicate that OH^−^ is not the promoter of CO_2_ reduction. In this study, the concentration of Na^+^ and OH^−^ was varied while keeping the other ionic content constant and the result was that the main supporting effect in the formation of C_2+_ products is caused by the sodium cation (Li et al., 2019a).

However, there are also disadvantages of using basic electrolytes, such as the already mentioned instability of imidazolium-based ionomers in alkaline environments (Kutz et al., 2017), but also the required stability of the catalysts and GDE. For example, C-based GDEs degrade after about 2 h when using a basic electrolyte (Dinh et al., 2018b). Furthermore, the dilution of CO_2_ in basic electrolyte leads first to the creation of HCO_3_^−^, followed by the conversion into CO_3_^2−^ (Leonard et al., 2020; Yang et al., 2020). This results in an indirect slowing down of the kinetics by initiating a shift of the pH value toward more neutral values and to the formation of barriers within the gas diffusion electrodes due to salinization, which in turn hinders the CO_2_ flow, promotes hydrogen formation and decreases the current density continuously (Endrődi et al., 2019; Yang et al., 2020). In addition, this storage of CO_2_ in the electrolyte can lead to an overestimation of the products FEs (Ma et al., 2020a), to conductivity losses within the system, as well as to energy efficiency losses in the overall electrolytic cell (Gabardo et al., 2019).

Finally, it should be noted that the amount of electrolyte used also influences the CO_2_RR performance. If there are larger amounts of electrolyte between the membrane and cathode, kinetics of HER is suppressed and separation of the liquid products is simplified, but larger ohmic losses occur within the cell, leading to higher cell voltages at moderate current densities (Chen et al., 2020b).

#### Local pH value

**Figure 9:**
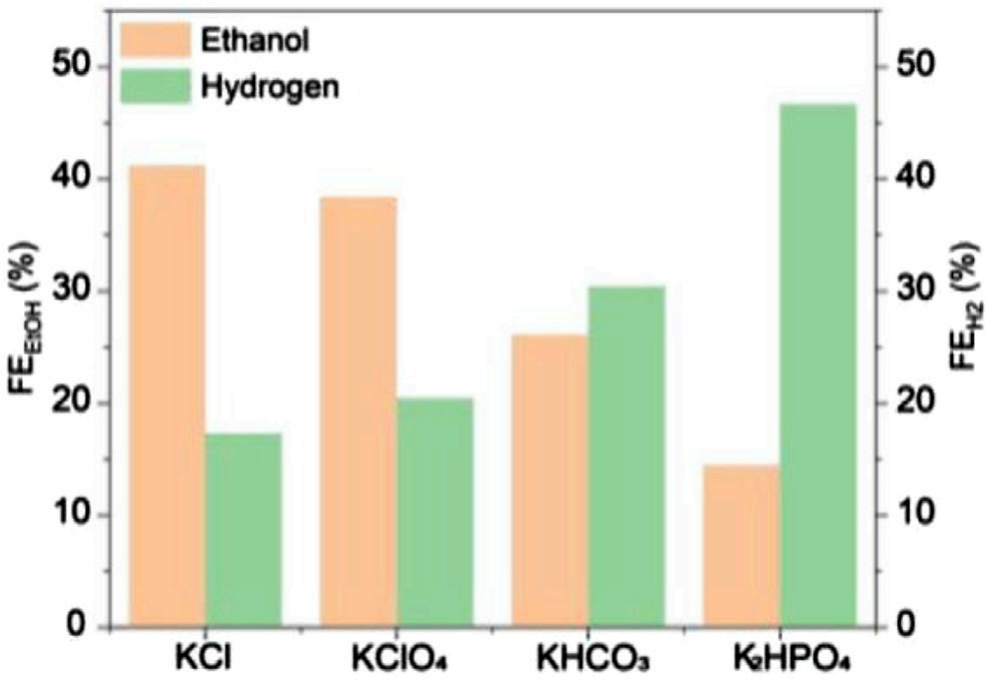
Comparison of FE_EtOH_ and FE_H2_ depending on chosen electrolyte solution with different buffering effects; H-type cell, at −1.1 V_RHE_ Reprinted by permission from John Wiley and Sons Ltd. (Kim et al., 2021).

The local pH value has a strong impact on the product distribution, as a more alkaline environment promotes CO and multicarbon product formation and suppresses HER and CH_4_ formation (Burdyny and Smith, 2019). Thus, the local pH has an influence on the energetics of the different products of the CO_2_RR. It has been observed that the pH in weak buffering solutions, such as KHCO_3_ or KCl, at the electrode can be shifted up to 6 units in the beginning of the electrolysis. The large pH difference can also cause difficulties in determining the equilibrium potential between the working and reference electrode correctly, which in turn influences the onset potentials. Within the catalyst layer, pH values above 12 may occur at current densities >200 mA cm^−2^. The use of acidic electrolytes in CO_2_RR is often regarded to be no alternative as hydrogen formation would become too dominant (Burdyny and Smith, 2019). However, recently Sargent and coworkers have shown CO_2_RR at 1.2 A cm^−2^ in 1 M H_3_PO_4_ yielding 50% FE of C_2+_ products, which is possible due to the drop of local pH during operation (Huang et al., 2021). Regarding the F-Cu catalyst investigated by Wang and coworkers, a correlation between the local pH value and the catalyst could be found (Ma et al., 2020b). It was shown that the local pH value at the electrode increases significantly in the order K_2_HPO_4_ < K_2_CO_3_ < K_2_SO_4_ due to the high concentration of OH^−^ produced during CO_2_RR, which cannot be buffered by electrolytes like K_2_SO_4_; however, the buffering of the pH value is better with, e. g. K_2_HPO_4_ (Lv et al., 2018a). At the same time, there is also an increase in C_2+_ formation in this order, which is more pronounced compared to the pure copper catalyst. In conclusion, it is however difficult to precisely estimate the extent of the pH influence on the catalyst (Ma et al., 2020b). A recent study by Jung and coworkers on Cu/Cu_2_O aerogel catalysts also shows that the use of electrolytes with lower buffer effect leads to higher FEs of ethanol at simultaneously lower FEs for HER (Kim et al., 2021). Thus, solvents with a higher buffering capacity should neutralize the OH^−^ generated during CO_2_RR and thus oppose the local pH effects. Figure 9 shows the FEs of EtOH and H_2_ as a function of the selected electrolyte, with an increase observed for ethanol in the order K_2_HPO_4_ < KHCO_3_ < KClO_4_ < KCl. Studies on an electrode with electrodeposited copper showed that the local pH at the oxidized copper electrode decreases from 10.4 to 9.3 with increasing negative applied potential ranging between −0.4 and −1.2 V and using 1 M KOH (Henckel et al., 2021). The decrease in pH is due to the formation of HCO_3_^−^, while at the same time, malachite is formed at the electrode at the beginning of the reduction of the copper oxide. Malachite shows highest thermodynamic stability between pH 8.0 to 10.5 and precipitates at the Cu surface of the electrode due to the carbonate-rich environment. These processes could influence the CO_2_RR product distribution. Thus, it should lead to higher Faraday efficiencies for the formation of ethylene than pure Cu foil. The subsequent further reduction of copper oxide and malachite finally leads again to a pH value of >11 (Henckel et al., 2021).

Recently, the local pH for pulsed electrolysis at CO_2_RR in CsHCO_3_ and LiHCO_3_, respectively, was determined by simulations (Kim et al., 2020a). These show deviating values depending on the applied potential. In the period of the pulse of −0.8 V, a high concentration of CO_2_ is present at the cathode surface; the local pH value of nine is low. When the potential is raised to −1.15 V, the pH also increases to 11, and the CO_2_ concentration decreases from the previous 31 mM to13 mM. It is likely that this results in additionally increased adsorption of ∗CO compared to ∗H, which is accompanied by increased C_2+_ selectivity with a concomitant decrease in HER (Kim et al., 2020a).

#### Electrolyte composition

Studies on the most appropriate electrolyte also considered the optimal choice of cations. With respect to alkali metals, the following trend was found for the selective formation of CO and EtOH (Karapinar et al., 2019) and for the current densities (Gao et al., 2018): Li^+^ < Na^+^ < K^+^ < Cs^+^. The difference is also very clear when comparing the FEs for the formation of ethanol using different electrolyte solutions. The use of a Cu-N-C catalyst achieved an FE for EtOH of 2% in CO_2_RR at −1.2 V in LiHCO_3_ solution, but 42% in CsHCO_3_ (Karapinar et al., 2019). If larger cations are used, these are less strongly hydrated, facilitating the adsorption on the surface of the catalyst (Karapinar et al., 2019; Kibria et al., 2019). The adsorption of those cations leads to a more positive potential of the outer Helmholtz layer (OHP), which in turn reduces the H^+^ concentration at the electrode, consequently lowering the extent of HER (Karapinar et al., 2019; Kibria et al., 2019; Lamaison et al., 2020). In addition, hydrated Cs^+^ ions near the catalyst surface can buffer pH changes and increase the amount of locally dissolved CO_2_ (Kibria et al., 2019; Jeong et al., 2020). Furthermore, the hydrated Cs^+^ ions would impose an electric field on the external OHP and thus promote C-C bond formation by coupling adsorbed ∗CO with ∗HCO (Jeong et al., 2020). With regard to the anions used, the best selectivity has so far been shown for OH^−^ in both CO and C_2+_ formation (Lv et al., 2018a; Kibria et al., 2019). Jiao and coworkers investigated the influence of the anions using KOH, KHCO_3_, KCl, and K_2_SO_4_ while keeping the K^+^ concentration constant (Lv et al., 2018a). The pH value in bulk was determined before and after electrolysis. It was found that KOH and KHCO_3_ showed hardly any changes in pH value in contrast to an increase of up to 4 pH units in the other two non-buffering electrolytes. The clearly best current densities for C_2+_ formation were obtained in KOH; in KCl and K_2_SO_4_, a high resistance was measured at higher overpotentials and a rapid overloading of the system occurred. The reason for the high resistance is probably the poor ionic conductivity of the membranes in these two electrolyte solutions (Lv et al., 2018a).

#### Halide additives

As already described with respect to the modification of catalysts with halides, the use of chloride, bromide, and iodide exerts a significant influence on the cell performance. Not only modifying catalysts but also adding halides to the electrolyte leads to considerable changes. For example, the addition of KX salts (X = Cl^−^, Br^−^ and I^−^) led to significantly increased current densities for the reduction of CO_2_ within an H-cell of plasma-activated copper catalysts in the order Cl^−^ < Br^−^ < I^−^ (Ma et al., 2020b). The FE of the C_2+_ products remained unchanged; current densities and formation rate for the products increased with increasing electronegativity of the halides. Investigations with KI addition showed that the increased activity for CO_2_RR takes place by accelerated hydrogenation of adsorbed CO intermediates (Dinh et al., 2018a). A significant rise in current density was also observed when CsI was added to a CsHCO_3_ electrolyte solution (Gao et al., 2018). It is assumed that the iodide adsorbs and thereby increases the roughness of the catalyst by quasi I^−^-induced nanostructuring. Thus, Cu^+^ is also stabilized by the iodide. According to the previous discussion concerning the cations' choice on the CO_2_RR, it can be observed that CsI, due to its larger cation, supports the CO_2_RR more than the addition of KI (Gao et al., 2018).

Because most studies currently focus on the formation of CO or C_2+_ products in general, it is necessary that further research on the influence of electrolyte solutions on the CO_2_RR to higher alcohols should be performed. Herein, preferential formation of C_2+_ products was reported frequently when using basic electrolyte solutions such as 1 M KOH and high local pH values. A disadvantage here, however, is the formation of carbonates in the GDE, which block diffusion pathways and facilitate HER. Contrary, recent results show that acidic electrolytes also have great potential for the formation of multicarbon products. A greater focus should therefore also be given to these systems, as it might be feasible to reduce carbonization effects in the GDE and enable a long-time stable system. In the context of zero-gap reactors, investigations should also be carried out using different solid electrolytes. In particular, materials should be found, which contribute to a low cell resistance, but at the same time are stable against the alcohols produced.

### Temperature

Another impact which was investigated on CO_2_RR is that of temperature. When using H-type cells, where the availability of CO_2_ at the cathode depends on the solubility of this gas in the used electrolyte, lower temperatures have been shown to facilitate a higher ratio of CO_2_RR to HER due to the better availability of CO_2_ (Ahn et al., 2017). Palmore and coworkers investigated the influence of the temperature on the CO_2_ reduction on polycrystalline copper. They reported that the temperature affects various electrolyte parameters like CO_2_ solubility, pH, resistance of the solution, and diffusion rate of the reactants. While the FE for methane increased with lower temperatures and peaked at 2°C, ethylene FE increased with higher temperatures reaching its maximum at 22°C. Activity for HER rose with increasing temperatures (Ahn et al., 2017). The effect of increased ethylene FEs with simultaneously lower methane FEs at elevated temperatures has also been described by other authors (Hori et al., 1986; Cook, 1988; Kim et al., 1988).

**Figure 10:**
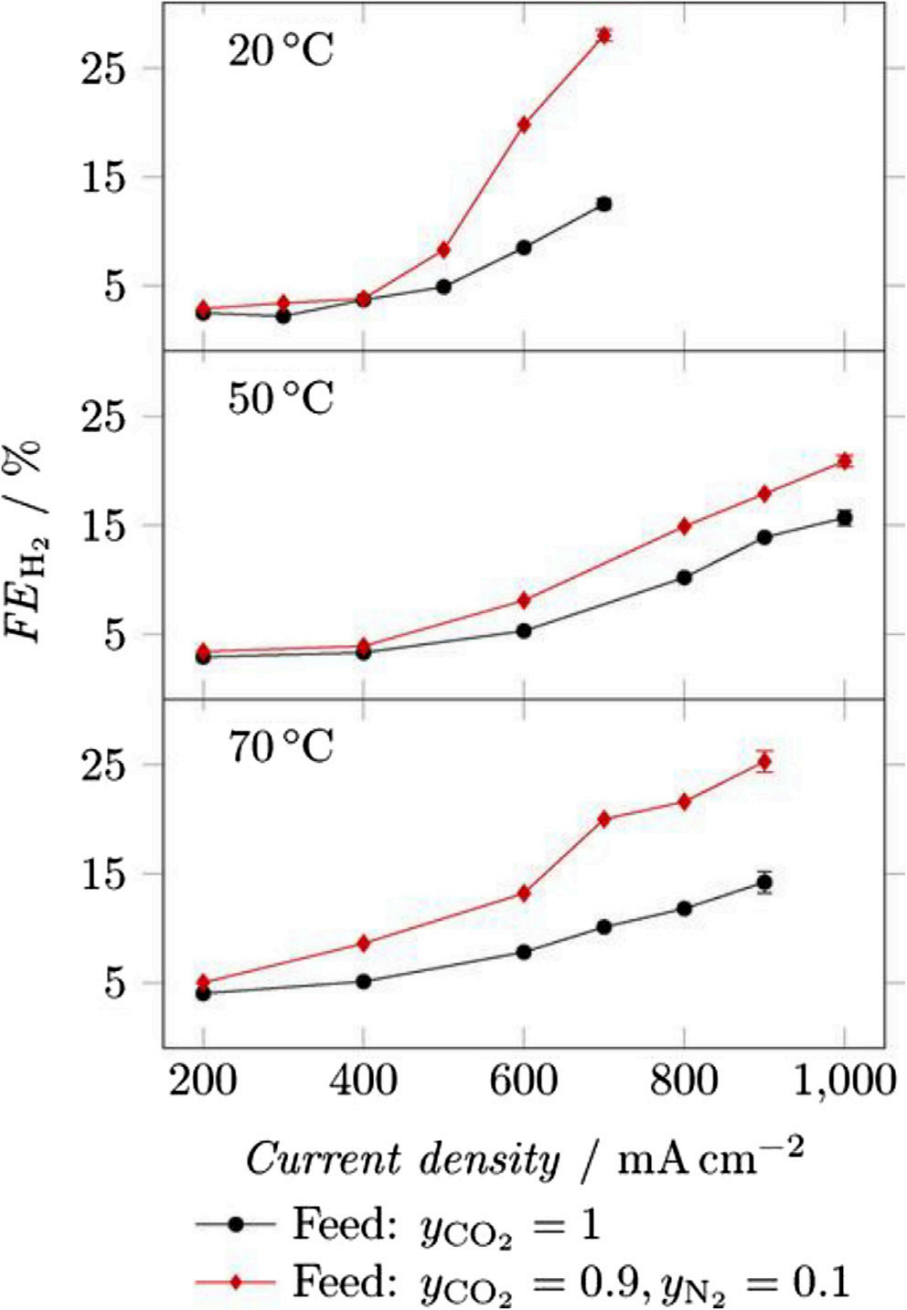
FE_H2_ at different temperatures, CO_2_ feeds, and current densities during CO_2_RR Reprinted from Löwe et al., 2019, *ChemElectroChem*, applying terms of CC BY license.

Besides H-type cells, investigation of temperature effects have been performed in flow cells. Klemm and coworkers researched the impact on the Sn catalyst-based CO_2_RR to formate in a liquid-phase flow cell. The optimum performance was observed at 50°C with over 80% formate FE at 1 A cm^−2^, while higher and lower temperatures led to increased HER (Figure 10). The increased HER at other temperatures than 50°C can be assigned due to the oppositional effects of reduced CO_2_ solubility and increased diffusion coefficients as well as faster reaction kinetics with increased temperature (Löwe et al., 2019). McIlwain and coworkers reported a reduction of the cell voltage by 1.57 V at 70 mA cm^−2^ during CO_2_RR to syngas, using a liquid-phase electrolyzer with an Ag-based GDE when the temperature is raised from room temperature to 70°C (Dufek et al., 2011). According to Sargent and coworkers, increasing the temperature to 60°C and the associated faster reduction kinetics and extended mass transport through the ionomer layer resulted in obtaining comparable Faraday efficiencies for C_2+_ products even at lower overpotentials (García de Arquer et al., 2020).

**Figure 11:**
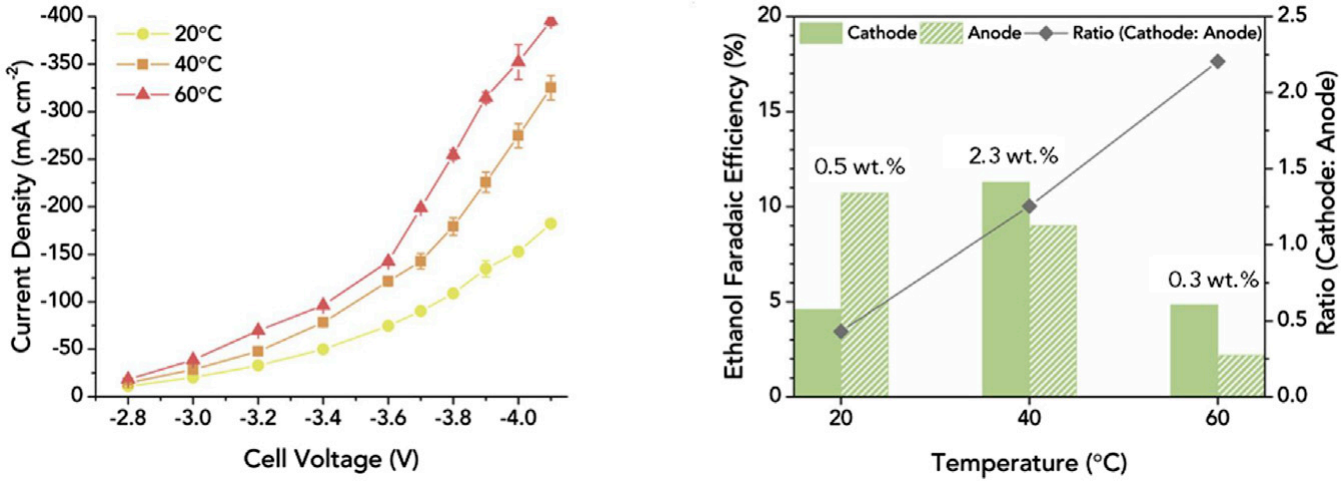
Impact of temperature on MEA during CO_2_RR on current density as well as on the FE of ethanol, recovered from both anode and cathode streams. Reprinted by permission from Elsevier (Gabardo et al., 2019).

In gas-phase electrolyzers, higher temperature can increase the electrochemical performance. According to Park and coworkers, reduction to formate on Sn nanoparticles increased more than 2-fold, when rising the temperature from 30°C to 90°C (Lee et al., 2018). Aricò and coworkers reported a significantly increased methanol production rate on a PtRu catalyst with higher temperatures, in a MEA-type setup, however, with overall low FEs (Sebastián et al., 2017). Sinton and coworkers also investigated the performance of their copper catalyst-based MEA electrolyzer at temperatures of 20°C, 40°C, and 60°C. An increase of temperature herein led to higher current densities for ethylene and hydrogen as well as higher FEs for the latter. Higher temperatures also increased the obtained ethanol output at the cathode side from 0.5 wt % at 20°C, peaking at 40°C with 2.3 wt %. This was attributed to an enhanced transport of water from anode to cathode side as well as increased vaporization of ethanol. The increased temperature was suggested to be a key factor in facilitating a highly concentrated output stream of liquid products (Gabardo et al., 2019). The influence of temperature on the MEA in terms of current density at different overpotentials as well as the described influence on ethanol yield is shown in Figure 11. The maximum temperature in gas-phase electrolyzers is limited due to a high rate of water crossover and the performance of the membrane (Kibria et al., 2019).

Initial studies have been carried out regarding temperature effects showing increased selectivity for ethanol at elevated temperatures. However, most studies addressing these reaction conditions deal with the formation of other products like formic acid or CO which means that there is still a lack on investigations showing the impact of various temperatures for a wider range of catalysts and electrodes for multicarbon alcohol production.

### Pressure

Another important parameter is the pressure of CO_2_. For cell types using CO_2_ dissolved in the electrolyte, the solubility of CO_2_ is increased with rising pressure, which results in higher current densities. As early as 1995, Sakata and coworkers investigated various metals at an elevated pressure of 30.4 bar in an autoclave H-type cell. The use of Ag, Au, Zn, Pb, and In for the CO_2_RR results in the preferential formation of CO and formic acid at standard conditions, and an increase in pressure also provided higher resulting current densities due to reduced overpotentials. For metals in groups 8–10 such as Fe, Co, Rh, Ni, Pd, and Pt, applying a pressure of 30.4 bar resulted in a shift in selectivity from HER to the formation of CO and HCOOH (Hara et al., 1995). A possible explanation for the change in selectivity is the facilitated desorption of CO under higher CO_2_ pressure (Hori and Murata, 1990; Kudo et al., 1993; Kibria et al., 2019). Using a dendritic Ag-Zn catalyst in an H-type cell containing 0.1 M CsHCO_3_, Vlugt and coworkers achieved a stable FE of over 90% for 40 h at 10 mA cm^−2^ and about −1.0 V vs. RHE. Pressurized measurements were performed in a single-chamber cell at 200 mA cm^−2^. Raising the pressure from 1 to 3 bar resulted in an increase in the partial current density for CO formation from about 30 mA cm^−2^ to 131 mA cm^−2^ at −2.0 V vs. RHE. A further increase to 6 bar even provided partial current densities of 188 mA cm^−2^ at −1.2 V vs. RHE (Ramdin et al., 2019). Mul and coworkers used Cu-NPs and KHCO_3_ to evaluate the resulting FEs in CO_2_RR under variation of pressure between 1 and 9.1 bar. The pressure increase raised the FE of ethylene from 10.8% to 43.7% while lowering the FE(CH_4_) from 21.3% to 1.8% and decreasing the HER. Although a lower local pH was calculated for higher pressures, the increased ethylene selectivity is associated with increased surface coverage of CO, which also causes higher yields of CO under pressure (Kas et al., 2015). Sakata and coworkers varied the pressure for the study of Cu electrodes in an H-cell between 1 and 60.8 bar, with an initial shift in selectivity from HER to hydrocarbons formation. The maximum was obtained at 40.5 bar, and as the pressure was increased further, the product spectrum shifted further toward CO and HCOOH (Hara, 1994).

Variation of pressure was also investigated in flow cells. So far, studies have mainly been performed on Ag-GDEs. Using these GDEs, a significant reduction in cell voltages was achieved by increasing the pressure (Hara, 1997; Dufek et al., 2012). McIlwain and coworkers combined the simultaneous increase of temperature and pressure (Dufek et al., 2012). Raising the temperature from 60°C to 90°C and increasing the pressure to 18.7 bar reduced the cell voltage from 4.01 V to below 3 V in the CO_2_RR to CO, with an FE(CO) of 82% (Dufek et al., 2012). Sinton and coworkers studied pressures from 1 to 7.1 bar using KOH electrolyte. The high pressure combined with 7 M KOH resulted in a low overpotential for reduction to CO of 300 mV at 300 mA cm^−2^ and an FE of almost 100%. Furthermore, the highest half-cell energy efficiency (EE) of 81.5% was achieved here compared with lower pressures (Gabardo et al., 2018). Recent studies on sputtered Ag-GDEs also show the effect of improved overall energy efficiency in CO_2_RR to CO of up to 67% at 202 mA cm^−2^, where the pressure was 50 bar and 5 M KOH was used (Edwards et al., 2020). In contrast, experiments by Schmid and coworkers on a silver-based GDE in a liquid flow cell showed no dependence of the FEs for CO when increasing the CO_2_ pressure from 0 to 25 bar. The CO_2_ pressure increase only caused a cell potential rise from 6 to 7 V, resulting in overall poorer energy efficiencies while no changes in FE were observable for CO formation (Krause et al., 2020).

In addition to CO_2_RR under pressure using aqueous electrolyte solutions, studies have also been conducted on the reduction of CO_2_ from supercritical CO_2_. CO_2_ behaves as a supercritical fluid meeting the critical pressure and temperature of 73.8 bar and 31.0°C (Span and Wagner, 1996). In this state, CO_2_ has the density of a liquid but the viscosity of a gas and is infinitely miscible with other gases (Abbott and Eardley, 2000; Melchaeva et al., 2017). Battistel and coworkers studied CO_2_ reduction on Cu electrodes in supercritical CO_2_ using acetonitrile as cosolvent and tetrabutyl-ammonium hexafluorophosphate to increase conductivity. In addition, protic solvents of different pH values were added to allow the formation of hydrocarbons and to influence the selectivity. The use of water and 1 M CsHCO_3_ resulted in FEs of 11.1% for ethanol and 7.5% for methanol. However, the overall FE was limited to 34%, possibly due to reoxidation at the anode and other sources of loss. In addition, corrosion of the Cu electrodes was observed (Melchaeva et al., 2017). Our group recently showed that under high pressure conditions in supercritical CO_2_, suppression of HER from an FE of 60% to below 8% is possible. The resulting shift in product distribution compared to measurements under ambient conditions led to current efficiencies of up to 66% for the formation of formic acid (Junge Puring et al., 2020).

Overall, the adjustment of process parameters could allow further optimization of electrochemical CO_2_ reduction, but the influence of temperature and pressure, especially for the reduction of CO_2_ to alcohols, has only been researched to a limited extent. In addition, aspects relevant for industrial implementation, such as long-term stability and integration into upstream and downstream processes, need to be evaluated.

## Conclusion & outlook

Finally, it can be stated that within the last few years, enormous efforts have been made regarding the design of catalysts for the electrochemical CO_2_RR to multicarbon products especially alcohols. The basis for these catalysts was almost exclusively copper-based systems. Overall, increasingly better Faraday efficiencies are being achieved for the formation of higher alcohols, with some exceeding 50% for C_2+_ alcohols (Karapinar et al., 2019; Chen et al., 2020a; Han et al., 2020a; Song et al., 2020; Wang et al., 2020b; Zhang et al., 2020a). Promising results were obtained by using alloys or bimetallic catalysts of Cu and, for example, Ag (Hoang et al., 2018; Li et al., 2019b; Dutta et al., 2020; Kim et al., 2020b; Martić et al., 2020; Zhang et al., 2020a), Zn (Ren et al., 2019; da Silva et al., 2020), or Pd (Rahaman et al., 2020). Likewise, catalysts with Cu and N-doped carbon showed encouraging results (Karapinar et al., 2019; Chen et al., 2020a; Han et al., 2020a; Wang et al., 2020b). These catalysts were prepared using different methods, of which sputtering (Li et al., 2019b, 2020; Wang et al., 2020b), electrodeposition (Dutta et al., 2020; Kim et al., 2020b; Rahaman et al., 2020; Kong et al., 2021), and precipitation conceivably followed by calcination, should be highlighted (Lv et al., 2018a; Zhou et al., 2018; Martić et al., 2019; Wei et al., 2020).

However, the selectivity does not solely depend on the catalyst, but also on the overall system. To achieve industrially relevant current densities, it is necessary to use flow cells or cells utilizing membrane electrode assemblies. H-cells, in which the CO_2_ transport to the catalyst is largely determined by its solubility in the electrolyte, should therefore be avoided in the future, especially because some studies show large differences in the product distribution for the same catalyst occur between H-cell and flow cell (Kibria et al., 2018; Gregorio et al., 2020; Wang et al., 2020a). In addition to the cell design, the design of the electrodes themselves is also of great importance. For a more detailed consideration of these two points, reference is made to the second part of the review *“Electrochemical CO*_*2*_ *reduction - The macroscopic world of electrode design*, *reactor concepts & economic aspects”*. There are few studies so far on the influence of temperature and pressure on the formation of multicarbon alcohols in CO_2_RR, but it was shown, for example by Sinton and coworkers, that a higher yield for EtOH could be obtained at a temperature of 40°C than at RT or at 60°C (Gabardo et al., 2019). Initial results are also available on the influence of pressure, even for using supercritical CO_2_ (Melchaeva et al., 2017).

Owing to the many influences, for example, from the process conditions, but also from the design of the electrodes and cells, further development of catalysts in terms of their selectivity is indeed sensible, but the following points in particular should be given more attention:

• Catalyst synthesis routes that are as simple as possible and do not involve particularly cost-intensive process parameters (such as high pressure) for insignificantly better selectivities (Siegmund et al., 2021) — better catalysts capable to reverse ethylene:ethanol selectivity are required
• A particular focus should be placed on the development of further tandem catalysts, as these materials have already shown promising results in terms of selectivity to multicarbon alcohols
• The targeted investigation and use of confinement effects, as already used for thermal catalysis (Mouarrawis et al., 2018)
• Research on ethanol and propanol selectivity in context of temperature increase should be investigated in detail
• Increasing the long-term testing and stability of the catalysts (for industrial implementation more than 1000 h tests are required (Masel et al., 2021))
• Reducing the cost of carbon capture by developing catalysts, electrodes, and cells that show good selectivities in terms of CO_2_RR even with lower CO_2_ concentrations
• Developing new electrode and cell designs (including membrane development) that allow for more selective and energy-efficient CO_2_RR (further discussion see “*Electrochemical CO*_*2*_ *reduction - The macroscopic world of electrode design*, *reactor concepts & economic aspects”*)
• Less catalyst testing in H-cells, because achievable current densities below 100 mA cm^−2^ are not industrially relevant
• Despite better CO_2_RR product distribution with regard to multicarbon products, turning away from KOH, because of the formation of carbonates and the oxidation of copper without applied potential—here more research regarding electrolyte influence on ethanol/propanol formation is essential
• Moving away from the use of liquid electrolytes through the application of MEAs to realize lower cell voltages and counteract flooding of electrodes, thus enabling higher long-term stability and continuous CO_2_RR

Investigation of mechanistic understanding, i. e. use of *in situ* technologies and operando methods like Raman or IR spectroscopy to realize better catalyst design resulting in higher selectivity toward multicarbon alcohols as products in CO_2_RR

### Limitations of the study

Owing to the enormous number of studies in the research field of CO_2_ reduction and the steadily increasing number of reports, it is not possible to know and cite every publication. It is pointed out that no author was specifically excluded. In order to give a comprehensive overview despite the high number of publications on the topic, two review parts have been written. This part deals especially with the catalysts/mechanisms/influences of the formation of higher alcohols during CO_2_RR; for other products please check other review papers.
